# Privacy-hardened and hallucination-resistant synthetic data generation with logic-solvers

**DOI:** 10.1093/bioinformatics/btaf600

**Published:** 2025-11-04

**Authors:** Mark A Burgess, Brendan Hosking, Roc Reguant, Anubhav Kaphle, Mitchell J O’Brien, Letitia M F Sng, Yatish Jain, Denis C Bauer

**Affiliations:** Australian e-Health Research Centre, Commonwealth Scientific and Industrial Research Organisation, Canberra, 2601, Australia; Australian e-Health Research Centre, Commonwealth Scientific and Industrial Research Organisation, Sydney, 2145, Australia; Australian e-Health Research Centre, Commonwealth Scientific and Industrial Research Organisation, Sydney, 2145, Australia; Australian e-Health Research Centre, Commonwealth Scientific and Industrial Research Organisation, Melbourne, 3052, Australia; Australian e-Health Research Centre, Commonwealth Scientific and Industrial Research Organisation, Sydney, 2145, Australia; Australian e-Health Research Centre, Commonwealth Scientific and Industrial Research Organisation, Sydney, 2145, Australia; Australian e-Health Research Centre, Commonwealth Scientific and Industrial Research Organisation, Sydney, 2145, Australia; Applied BioSciences, Faculty of Science and Engineering, Macquarie University, Macquarie Park, 2109, Australia; Applied BioSciences, Faculty of Science and Engineering, Macquarie University, Macquarie Park, 2109, Australia; Australian e-Health Research Centre, Commonwealth Scientific and Industrial Research Organisation, Adelaide, 5000, Australia; Department of Biomedical Informatics and Digital Health, School of Medical Sciences, University of Sydney, Sydney, 2050, Australia; The University of Adelaide, Australian Institute for Machine Learning, Adelaide, 5000, Australia

## Abstract

**Motivation:**

Machine-generated or synthetic data is a valuable resource for training artificial intelligence algorithms, evaluating rare workflows, and sharing data under stricter data legislations. However, current statistical and deep learning methods struggle with large data volumes, are prone to hallucinating scenarios incompatible with reality, and seldom quantify privacy meaningfully.

**Results:**

Here, we introduce Genomator, a logic solving approach (SAT solving), which efficiently produces private and realistic representations of the original data. We demonstrate the method on genomic data, which arguably is the most complex and private information. We benchmark Genomator against state-of-the-art methodologies (Markov generation, Wasserstein Generative Adversarial Network and Conditional Restricted Boltzmann Machines), demonstrating a 40%–530% accuracy improvement and 57%–172% higher privacy. Genomator is also 3–100 times more efficient, making it the only tested method that scales to whole genomes. We show the universal trade-off between privacy and accuracy, and use Genomator’s tuning capability to cater to all applications along the spectrum, from provable private representations of sensitive cohorts, to datasets with indistinguishable pharmacogenomic profiles. Demonstrating the production-scale generation of tuneable synthetic genomes hold great potential for balancing underrepresented populations in medical research and advancing global data exchange.

**Availability and implementation:**

Genomator is available at https://github.com/csiro/genomator.

## Graphical Abstract

**Graphical Abstract:**
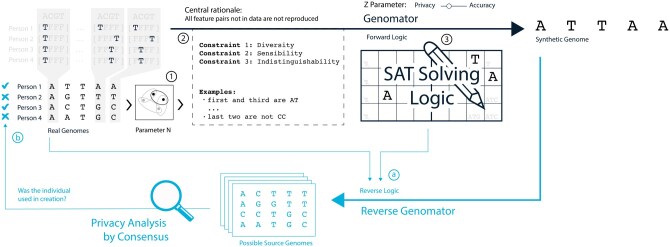

## 1 Introduction

Synthetic data are emerging as a valuable resource in the medical sector (Gonzales *et al.* 2023). This is because stricter data sharing and privacy regulations imposes limitations on the exchange of real data (Rehm *et al.* 2021). Furthermore, synthetic data can be used as a reference dataset to test bioinformatics software (Souche *et al.* 2022). Synthetic genomes are of particular interest due to the high cost of obtaining data through sequencing and the risk of exposing personally identifiable or sensitive information when sharing real genomic data. Use cases for synthetic genomes include (i) ‘digital twin’ dataset (Emmert-Streib and Yli-Harja 2022) where population-specific genomic information is reproduced without identifying any one individual specifically (Bonomi *et al.* 2020), (ii) ‘Truth Challenges,’ where synthetic genomes are spiked with difficult to identify variants for grading pathology providers or software evaluation (Olson *et al.* 2022), or (iii) for boosting sample representations in the ‘long tail’ of human genetic diversity and disease states (Gonzales *et al.* 2023).

The latter use case is highlighted by Gartner, who expects synthetic data to ‘completely over-shadow real data in AI models by 2030’ (Ramos and Subramanyam 2021). To ensure the synthetic data sources can be used for such a purpose, various conditions need to be met. Particularly, the generative algorithms need to adequately reflect the diversity and complexity of features (or patterns) of the real data (Chen *et al.* 2021). In addition, privacy must be preserved where, for genomics, exposing even a single nucleotide can reveal sensitive information, e.g. breast cancer risk (Claus *et al.* 1994), or expose the identity of an individual through a rare private variant (Von Thenen *et al.* 2019). As such, generative algorithms need to be explainable, so the origin of the generated nucleotide and its risk can be tracked. Lastly, fit-for-purpose algorithms in the genomics space need to be resource efficient (Three steps for businesses to make AI data and compute more sustainable—OECD.AI) and scale to the dimension of genome applications.

Traditional approaches for creating synthetic genomic information generate novel haplotypes by resampling from reference genomes, based on a stochastic model that simulates the underlying processes of coalescence, recombination, and mutation. Examples are HapGen2 (Su *et al.* 2011) and Hapnest (Wharrie *et al.* 2022). However, these methods require additional information (e.g. genetic parameters related to evolutionary history such as recombination rate, allelic age, mutation rate) and can hence be biased if the wrong assumptions were made. Furthermore, this information is typically not available for large genomic cohorts.

More recent methods developed by Yelmen *et al.* (2021) are unsupervised, including approaches like Markov Models, Generative Adversarial Networks (GANs), and Restricted Boltzmann Machines (RBMs). Their most recent paper (Yelmen *et al.* 2023) introduced Wasserstein GAN (WGAN) and Conditional RBM (CRBM) methods as extensions of their previous approaches. These approaches are designed to generate larger scale genome segments by machine learning over local information and by conditionally generating and adjoining smaller generated segments respectively. However, the underlying ML approaches are resource intensive and cannot scale to whole genome applications in their current form.

In this manuscript we present Genomator, a resource efficient SAT solving application for *the de-novo* creation of synthetic data. SAT solvers are a class of algorithms to solve mathematical and combinatorial problems that involve the (SAT)isfaction of constraints among Boolean variables (Biere *et al.* 2021). As such, Genomator can deductively generate synthetic data that possesses the desired qualities of the real data, while its logic engine ensures explainability and scalability to whole genome applications.

We systematically compare the accuracy and privacy of Genomator against the current state-of-art approaches (Markov chain generation, Wasserstein Generative Adversarial Networks, and Conditional Restricted Boltzmann Machines) and demonstrate its utility in the pharmacogenetics space.

## 2 Materials and methods

### 2.1 Datasets

For evaluating population-structure we used the 65K single nucleotide polymorphisms (SNPs) from across the genome as selected by Yelmen *et al.* (2023) for the 2504 samples in the 1000 Genomes Project (1KGP) (Auton *et al.* 2015). Principal component analyses (PCA) were conducted using the SciKit-Learn PCA algorithm and plotted using MatplotLib python libraries.

For evaluating the runtime scalability, we generated data over increasing sections (excluding sex chromosomes) from the latest release of the 1KGP project using the first 400 of the 3201 samples, with filtering for the retention of variants with a minor allele frequency (MAF) greater than or equal to 0.01, the exclusion of variants showing deviations from Hardy-Weinberg Equilibrium (HWE) with a significance threshold set at 1e-06, and the removal of duplicate variants from the dataset.

For the calculations of pharmacogenetic SNP allele frequencies, chromosomes 10 and 16 were selected as they house three important pharmacogenetic SNPs: *rs*4244285, *rs*4986893, and *rs*9923231. We used the latest release of the 1KGP project, including all 3201 samples, without applying Hardy-Weinberg Equilibrium (HWE) filtering. A total of 1000 synthetic genomes were generated for the analysis and their ‘ethnicity’ was inferred based on the clustering of their PC projections with the 1KGP annotated ‘Super Populations’.

All filtering was done using PLINK2 (Chang *et al.* 2015).

### 2.2 Software tools

**Figure 1.**
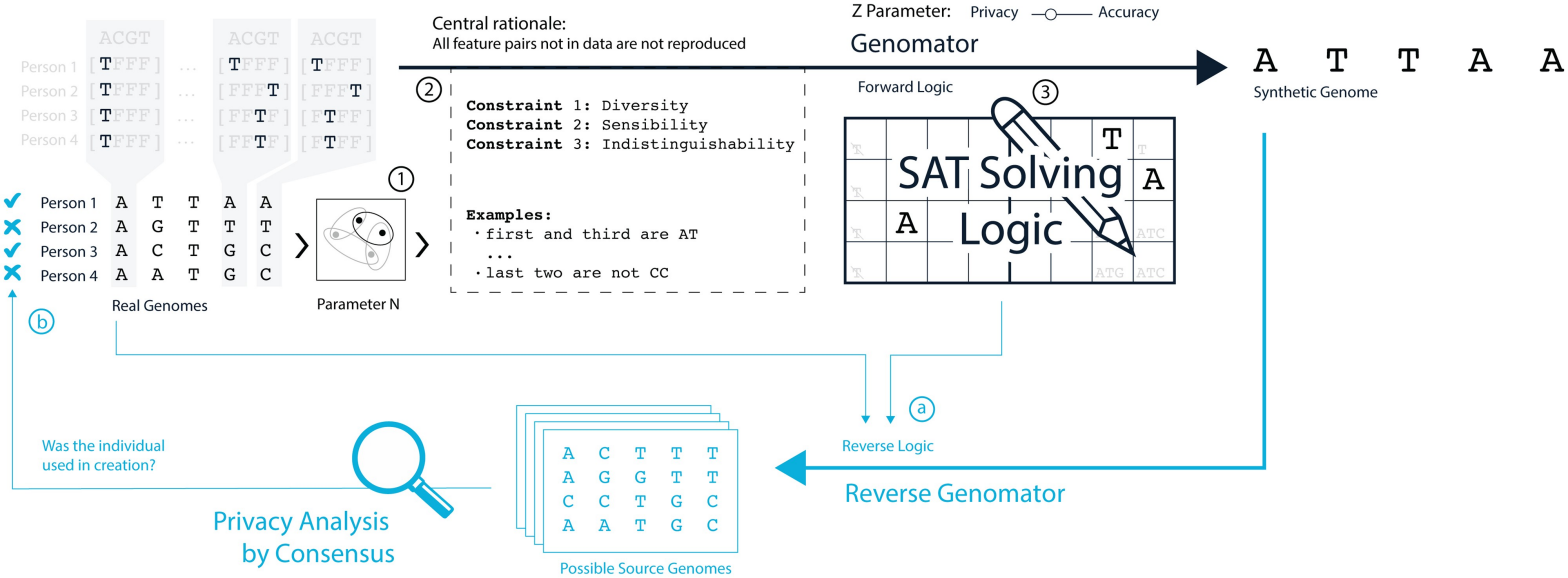
An illustration of Genomator (black) and Reverse Genomator (blue). From a cohort of real genomes, we first create overlapping clusters of size *N* (1). From a randomly chosen cluster, constraints are formed based on observations in the data (2).

*Genomator* constructs synthetic data by formulating a logical problem and solving it using a SAT solver as illustrated in Fig. 1. Genomator first clusters the input dataset of real genomes (specified in VCF format) into *N* clusters (by Hamming distances). It then randomly selects a cluster and uses a SAT solver (Ignatiev *et al.* 2018) to generate realistic synthetic data by ensuring that almost all pairs of features not observed in the cluster are also not reproduced in the synthetic genome. To create the next synthetic genome, a new cluster is chosen to repeat the process.

The degree to which the synthetic data is replicated can be modulated. For example, Genomator might only consider a random subset of feature pairs during the generation of synthetic data, and parameter *L* determines the size of this fraction. Similarly, setting a higher cluster size (*N*) increases the diversity of features observed within a cluster, which results in less constrained synthetic data. Lastly, the privacy parameter *Z* controls the degree to which rare feature pairs are attenuated as it filters out features that occur with a frequency at-or-below the randomized parameter *Z*. For our experiments, this means that for every feature pair that is observed only once in the real data (rare), *Z* is chosen to be 1 with 33% probability (meaning it can be replicated in the synthetic data), or 0 with 66% probability (meaning it will be suppressed). A shorthand notation of this is *Z* = 0.5, where ‘*Z* = α’ denotes *Z* being a random integer parameter resulting from a rounding down of a uniform random variable in the interval [0, α + 1). The interplay between *L*, *N* and *Z* hence controls how conservative the resulting synthetic data is—with implications on accuracy and privacy.

Except where noted otherwise, a cluster size of *N* = 150, exclusion probability of *L* = 0.99, and *Z* = 0.5 were used in all experiments. For the experiments where *Z* is varied (Section 3.3,3.4), a notation ‘*Z* = 3’, is shorthand for *Z* = 0 with probability 25%, *Z* = 1 with probability 25%, *Z* = 2 with probability 25% and *Z* = 3 with probability 25%, and *Z* = 4 with probability 25%, etc. For calibration, please see notes in Sections 9 and 10, available as supplementary data at *Bioinformatics* online.

*Reverse Genomator* conducts Genomator’s logical reasoning in reverse by constructing a different logical problem and solving it using a SAT solver. Reverse Genomator considers synthetic genomes generated using Genomator, and then resolves possible combinations of input clusters which Genomator could have used to generate them. In this way, if there is no consensus of membership among the possible input clusters that Reverse Genomator generates, an indication of the privacy afforded by Genomator is given, as the synthetic genomes could have been generated many different ways with-or-without the presence of any particular real genome in the input cluster.

*Markov Chain* method as implemented by Yelmen *et al.* (2021) and, except where otherwise noted, was conducted with a window size parameter of 330 variants. Additional details are noted in Section 3.1, available as supplementary data at *Bioinformatics* online.

*Wasserstein Generative Adversarial Network* (WGAN) as implemented by Yelmen *et al.* (2023) and documented in their repository with training was conducted to 1700 epochs. Additional details are noted in Section 3.2, available as supplementary data at *Bioinformatics* online.

*Conditional Restricted Boltzmann machine* (CRBM) implementation from Yelmen *et al.* (2023) as documented in their repository with training was conducted to 20 000 epochs. Additional details are noted in Section 3.3, available as supplementary data at *Bioinformatics* online.

### 2.3 Accuracy calculation

*Wasserstein distance is used* for analyzing the consistency between datasets. We used the Sliced Wasserstein distance algorithm in the Python Optimal Transport library (POT: Python Optimal Transport–POT Python Optimal Transport 0.9.3 documentation; Flamary *et al.* 2021). In Section 3.1, to quantify the differences in the PCA of the synthetic versus real data on the Yelmen 65K SNP dataset, we calculated the Wasserstein distance on the first two principal components between synthetic and real. In Section 3.4, we evaluated the Wasserstein distance on the data itself, hence calculated the Wasserstein distance across all dimensions between input and synthetic data and reported percentage error as proportion of the maximal of 800 haplotypes.

*LD correlation error* used for Fig. 3 was calculated from pairwise LD evaluated using the Rogers-Huff r-squared method (Rogers and Huff 2009), which is part of the Scikit-Allel Python package. LD reproducibility between synthetic and real dataset is quantified by average LD square error, obtained by subtracting the pairwise synthetic LD values from the dataset true LD relationships and squaring the result and averaging. We evaluated synthetic data LD reproducibility generated from a training set against a test set—produced by equal split partition of datasets—and calculated this difference in LD between synthetic and real data for all SNP pairs within window sizes averaged across the tested genome. We systematically test window size ranges from 50 000 to 250 000 base pairs in equal increments.

### 2.4 Privacy calculation

*Attribute inference-based privacy* scores and links the synthetic genome against the real genomes to quantify the re-identification risk and how much information an attacker could gain about real individuals. It emulates an inference attack scenario where an attacker ‘fills in’ a target’s missing SNP data from the closest synthetic data. We split the original genome dataset into two random subsets and generate synthetic data from each. Next, we compute the median closest Hamming distance between the input sequences and to the synthetic dataset created from the subset containing the input sequence (in-data) and the subset that does not contain it (out-data). The difference between the in-data and out-data distances reflects privacy by the additional likelihood that an attacker correctly identifies a target’s SNP attributes from synthetic data because it was created using that target individual’s data. This approach reflects Giomi *et al.* (2023) (inference attack) except baselining performance against synthetic data from a hold-out set, instead of random imputations.

*Private and fictitious quadruplet revelation* measures privacy by how likely rare combinations of SNPs are reproduced in the output—and thus how easily the output could be used to infer individuals with rare trait combinations in the input. To do this, we randomly selected SNP combinations in groups of four (quadruplets) and assessed if they are private—meaning the combination occurs exactly once in the original data—or fictitious, meaning it does not appear at all in the original data. We continued sampling until we have 100K fictitious and private combinations. Next, we generated 2500 versions of synthetic data and determined how many of the 100K SNP combinations occurred across these 2500 datasets to compute the likelihood for each category, i.e. private or fictitious. This reflects Giomi *et al.* (2023) (‘singling out’) except identifying unique combinations among the input data for comparison to the synthetic data, instead of *vice versa*.

*Exposure risk* deduces privacy directly and is unique to our logic-based approach. We randomly selected *G* SNPs and *M* individuals from the 65K SNP dataset. We then generated 500 distinct plausible input combinations (of same size *M*) for a synthetic genome created from the same cohort using Reverse Genomator. We then identified individuals present in all 500 combinations and labelled them as *exposed*. This process is repeated across multiple iterations, where the event that any individual is exposed is modelled as a binomial variable. The exposure risk is the mean of this binary variable, measured as the proportion of iterations where an individual appears in all reconstructed combinations, with confidence intervals calculated based on the binomial model. Please note, in a hypothetical scenario where an attacker is looking for ‘likely enough’ cases, a Bayesian analysis can be performed as given in Theorem 1, available as supplementary data at *Bioinformatics* online.

## 3 Results

### 3.1 Accuracy: SAT-solvers replicate both genome-wide and local genomic structure

We evaluate the ability of the methods to produce synthetic data that captures the higher-order complexities of genomic data. We ran a PCA on the synthetic data generated from the Yelmen 65K SNP dataset to measure how accurately each method reproduces the well-known ‘V’ shape of the underlying population structure. We plot the synthetic data against a hold-out set of the real data.

**Figure 2.**
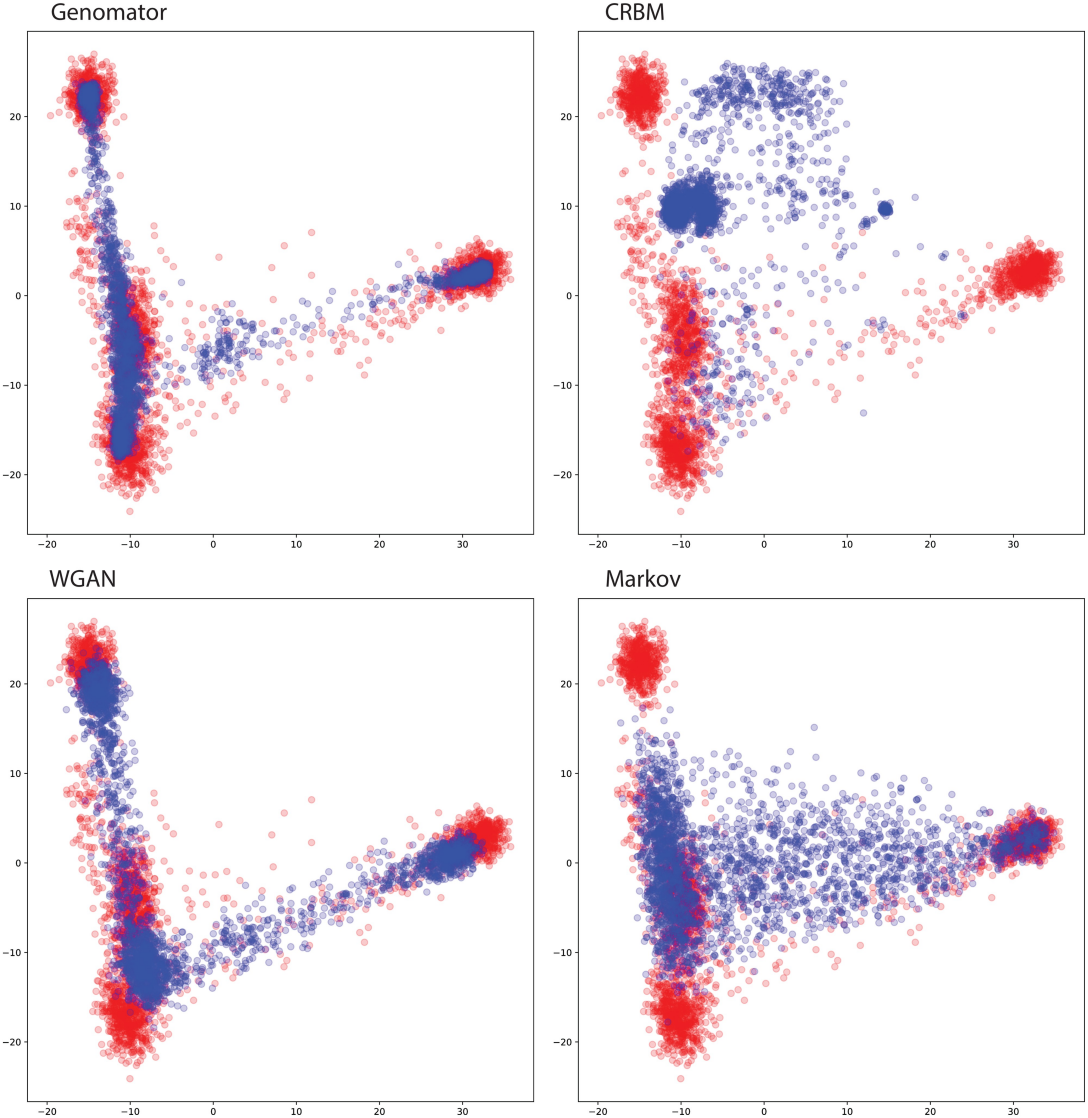
Principal Component Analysis (PCA) of generating synthetic data using different techniques on the 65K SNP dataset, showing components of first two principal components of the real data (red) and the 2500 generated synthetic data (blue) projected onto those components.

Figure 2a shows that Genomator reproduces the population structure accurately (Wasserstein score 2.296) whereas Markov and WGAN produce synthetic data that are dispersed and slightly shifted from the real data (Wasserstein scores 7.446 and 3.234 respectively). CRBM performs poorly despite using implementation and parameters as reported (Yelmen *et al.* 2023) (Wasserstein score 14.514), showing clustering of synthetic samples and the loss of the characteristic ‘V’ shape. In this context, Genomator is demonstrated to be 40%–530% more accurate.

We also evaluate the methods’ abilities to replicate genome correlations as these are of particular importance to medical and research applications (Sved and Hill 2018). We produce 2500 synthetic genomes for each method on the 65K dataset and compared the LD patterns constructed to the original dataset. We quantify the error in capturing LD patterns for all methods by calculating the average square error between the real and reproduced LD with increasing window sizes across the gene.

**Figure 3.**
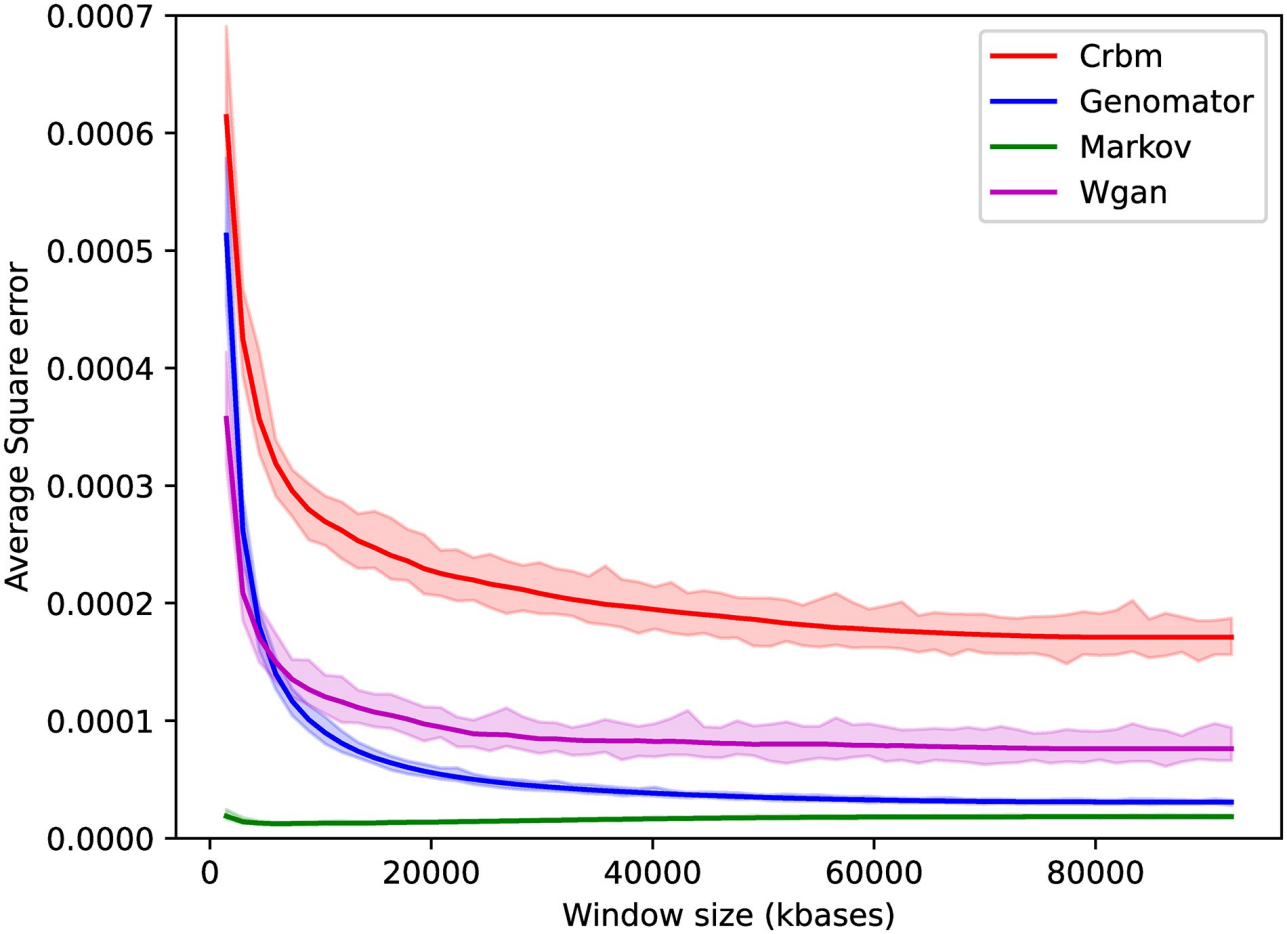
The average square error in reproducing the LD pairwise relationships between pairs of variants, averaged within a window size of base pairs, between the synthetic dataset created with a training set against a hold-out set. 95% confidence bands calculated by bootstrapping method.

As shown in Fig. 3, Markov Chain returns the best result (average square error of 0.000018), followed by Genomator (0.000031), compared to GAN (0.000076) and CRBM (0.00017). While Genomator is at least twice as good as GAN and CRBM, Markov best reproduces local correlation patterns between SNPs. This is because Markov generates SNP information directly from correlations in a small *n*-sized window, while the other methods also aim to capture long-range information as demonstrated by Fig. 2.

### 3.2 Efficiency: SAT-solvers enable scaling to full genomes

We evaluate the runtime of generating a synthetic genome from each of the methods, including all training and processing, but discounting initial input and final output file operations as they would be the same between methods. We report the runtime over generating increasing number of SNPs, from 100 to the whole genome of 11.7M SNPs, from 400 samples of the 1KGP. We ran all tests with Intel Xeon(R) CPU Platinum 8452Y, 2.00 GHz, with 80 GB of RAM and allowed up to 3.5 days of compute time. CRBM and WGAN methods also utilized a GPU Nvidia H100 94GB graphics card while Markov and Genomator utilized a single core.

**Figure 4.**
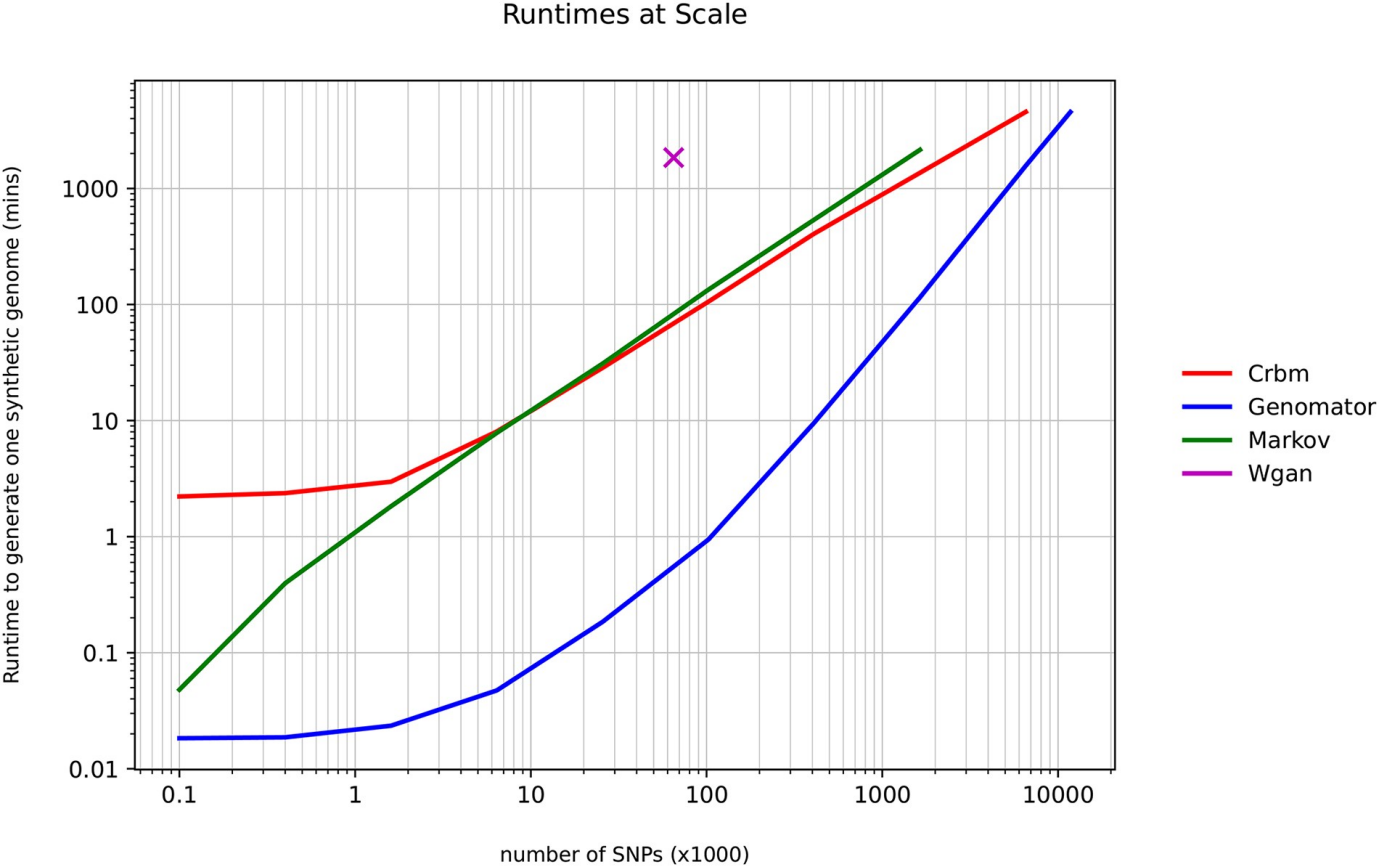
Runtime to create a synthetic datum from increasing sized input data up to 11.7 million SNPs—the full number of SNPs from chromosomes 1–22 with MAF > 0.01 filtering, 400 samples, from 1KGP project.

The data in Table 1 and Fig. 4 show that Genomator is the fastest of the tested methods, particularly at generating smaller genome fragments at 100 times faster, and with larger genome fragments between 3 and 10 times faster. The WGAN architecture reported by Yelmen *et al.* (2023) is tailored to the 65K dataset and hence represented as a single point in the figure. Importantly, Genomator is the only method that scales to the full 11.7M SNPs.

**Table 1.** Runtimes in seconds of each of the methods against number of SNPs.

SNPs	Genomator	CRBM	Markov
100	1.10	133.11	2.88
400	1.12	142.22	23.83
1600	1.41	178.38	109.49
6400	2.84	482.83	470.26
25 600	11.07	1709.55	1856.99
102 400	56.64	6347.69	8055.63
409 600	576.98	24 480.52	32 301.40
1 638 400	6921.72	81 859.87	129 146.67
6 553 600	94 212.70	274 069.62	
11 757 483	283 097.15		

While SAT-solvers are fast at capturing the input data, they require a fixed amount of resources per individual to generate new synthetic genomes compared to CRBM or WGAN, and this linear relationship is shown in Section 8, available as supplementary data at *Bioinformatics* online. Table 2 shows the increase in resources consumed for 120 and 2500 genomes, respectively, on the 65K dataset. While for 120 genomes, Genomator performs comparably, the break-even point tips in favor for ML methods when generating more genomes.

**Table 2.** Runtimes for generating a number of genomes on the 65K dataset for different methods.

No. of genomes	Genomator	CRBM	Markov	WGAN
120	14 183.39	44 591.06	38 000.31	103 850.24
2500	314 526.90	47 679.91	83 052.09	104 401.54

### 3.3 Privacy: SAT-solvers are hallucination resistant and private

We measure privacy using two approaches (attribute inference, private quadruplet revelation). These methods provide an alternative to similarity-based privacy evaluations, as used by Yelmen *et al.* (2023) who use nearest neighbor adversarial accuracy (Yale *et al.* 2020), which have since been criticized by Stadler *et al.* (2020) for their shortcomings around quantifying privacy risks.

Adapting the approach from Giomi *et al.* (2023), we simulate an attribute inference attack by first splitting the 65K SNP dataset into two equal and similar subsets by random selection and generating 120 synthetic genomes from each. We calculated the ‘in-data’ distance as the median nearest neighbor distance between the real data points and the synthetic data generated from the subset including those points, forming an accuracy measure. The ‘out-data’ distance was calculated as the median nearest neighbor distance between the real data points and the synthetic data generated from the subset not including them (Fig. 3, available as supplementary data at *Bioinformatics* online). The difference between in-data and out-data distances can then be interpreted as a measure of privacy, quantifying how much more likely a hypothetical attacker could guess SNP data of a target individual due to information leakage in the generation process.

**Figure 5.**
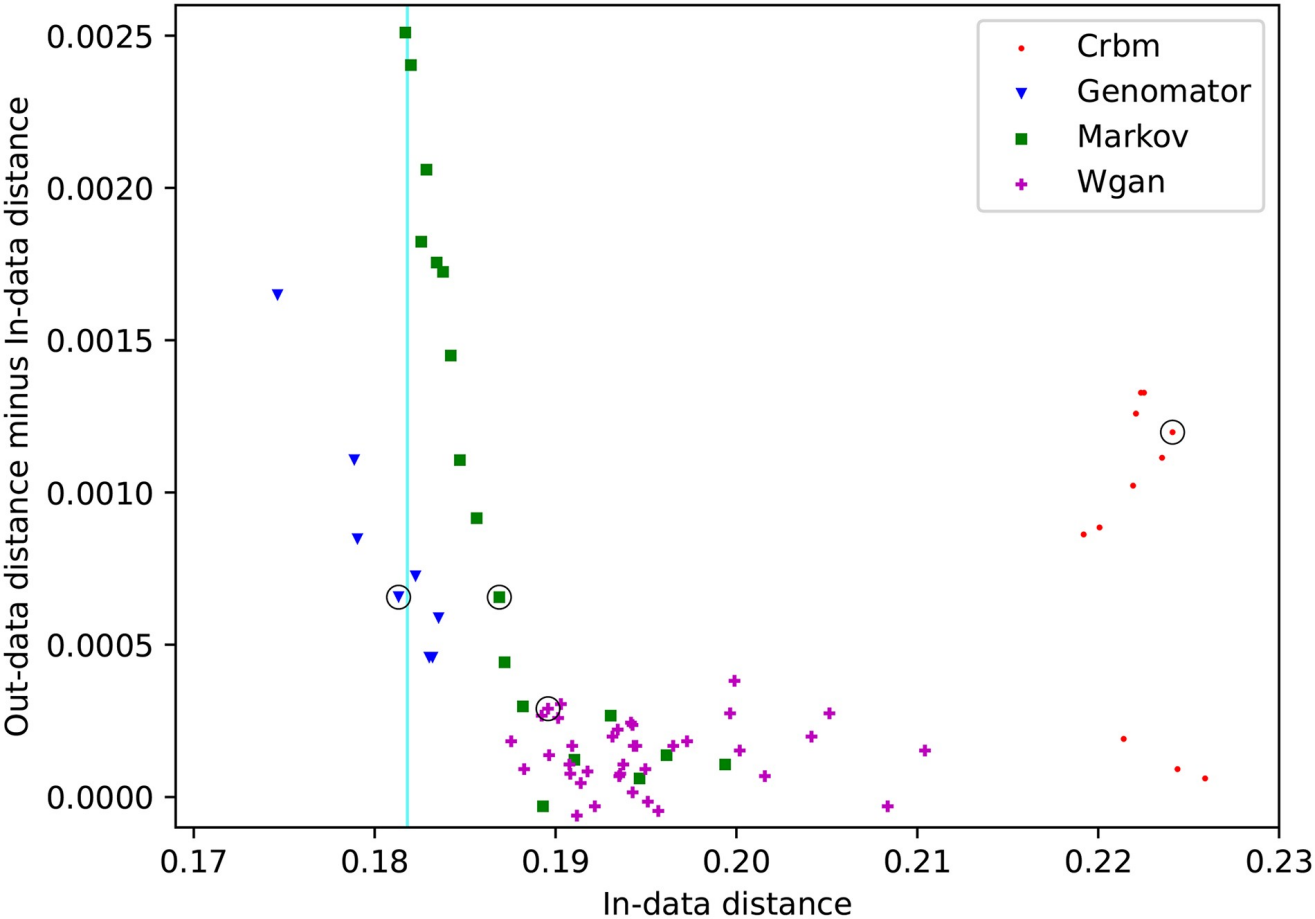
Results of SNP inference experiment on 805 SNP data for different methods. The ideal outcome is a perfect fit (in-data = 0) and no difference between in- and out-data distance.

Figure 5 reports these distances for each method using a range of parameter settings (e.g. CRBM for 2000–20 000 epochs of training, Markov with window-size 10–690, WGAN trained to 170–1700 epochs, Genomator *N* = 50–250 and *Z* = 0.5–2.5). Each method creates a unique ‘frontier’ that traces the trade-off between accuracy and privacy leakage. Genomator and Markov create a ‘frontier’ along the privacy axis while other methods are more static in their capacity to fit to the input data resulting in less flexibility in the outcome.

The vertical line indicates the average in-data distance (accuracy) from the real data to itself. We selected the Genomator parameters that were the closest to the line and offered the best privacy (circled in Fig. 5) for Genomator’s default parameters in this paper. Section 10, available as supplementary data at *Bioinformatics* online details the selection process for the optimal parameters for each dataset. For Markov’s default parameter, we selected the settings with the same level of privacy as Genomator. For WGAN and CRBM the results used (encircled) are the ones trained to the maximal epochs.

Our second privacy measure drills down into the privacy dynamics further by quantifying how often ‘private’ SNP combinations leak into the synthetic data output. These are sets of SNPs that are only seen in one individual in the input dataset and hence replicating them might expose uniquely identifiable features of that individual and facilitate identification. We compare this to the ‘hallucination’ rate at which fictious SNP combinations (that are not seen in any individual) are created to quantify the balance between accuracy and privacy.

In the synthetic versions of the 65K data generated using the methods, we compute the likelihood that private and fictitious combinations of SNPs appear in the output dataset. To do this, we sampled 10 000 random quadruplets and assessed whether they were fictious or private, counting the respective occurrences for each method to obtain the likelihood. The ideal method produces fewer privacy-revealing combinations, while avoiding creating unseen combinations that are potentially not viable in humans.

**Figure 6.**
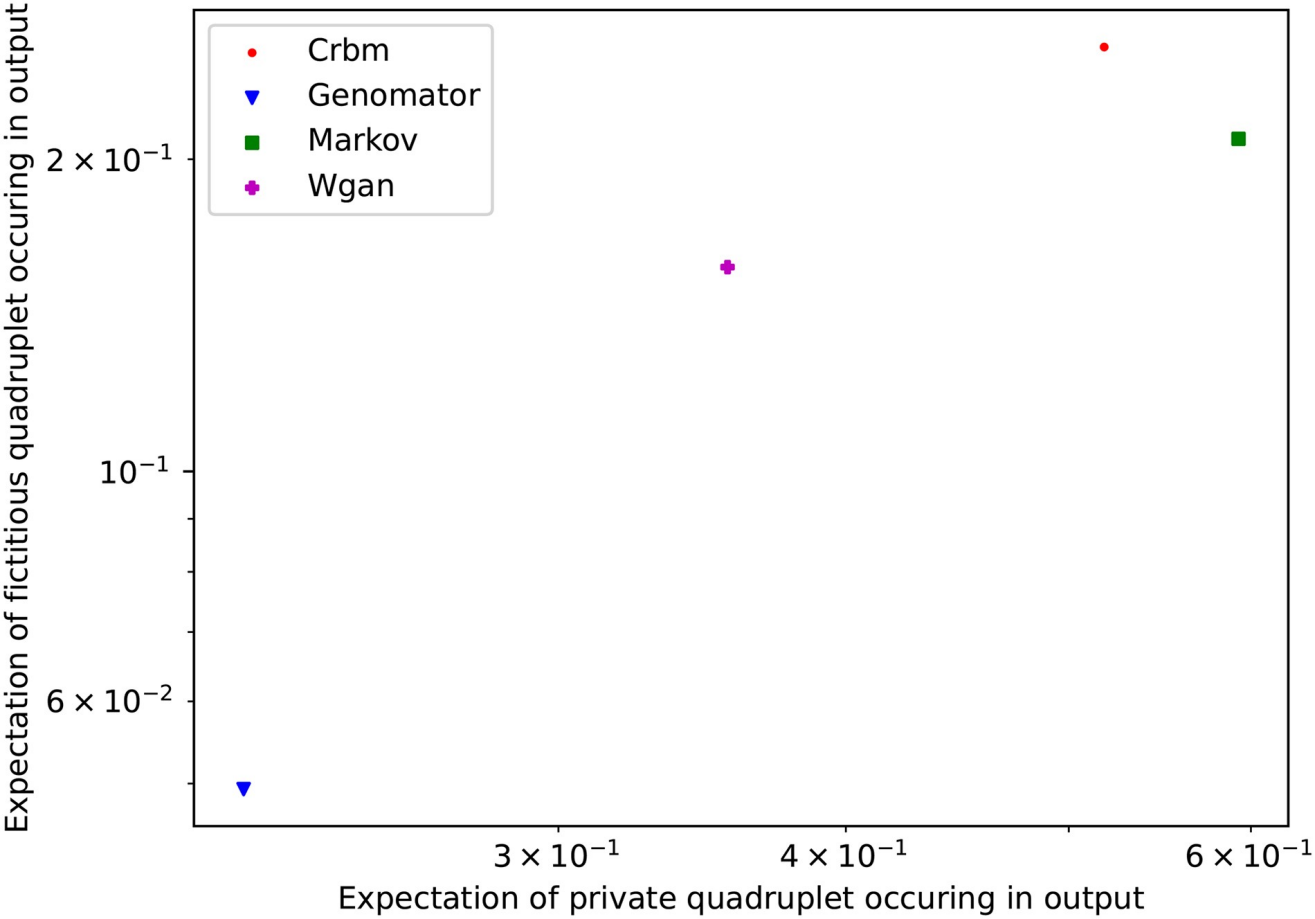
For each method, the likelihood of quadruplet combination of SNPs in the output being ‘fictitious’ versus private. Where a fictitious quadruplet is one that is not featured in the input dataset and a private quadruplet is one that is featured in exactly one individual in the input dataset.

Figure 6 shows that Genomator is the most hallucination resistant: 228% more than WGAN, 338% more than Markov, and 435% more than CRBM. Genomator is also more resistant to private combination leaking: 57% more than WGAN, 131% more than CRBM and 172% more than Markov—see Section 6, available as supplementary data at *Bioinformatics* online.

### 3.4 Novelty: Genomator can customize the level for accuracy-privacy trade-off

Here we measure privacy absolutely compared to the previous section which measured privacy by proxy. Unlike other approaches, such as WGANs and CRBMs, SAT-solvers are logic-based approaches which allow reverse inferences. We hence developed Reverse Genomator, which identifies for any synthetic genome, the logical space of all possible combinations of subsets of input data that could have been used to generate it (given the full information about Genomator and a dataset from which Genomator’s cluster input is selected from). Individuals who appear in all these subsets are deduced to have been used as input and are considered ‘privacy exposed’ (see Section 2.4).

As noted in the previous section, there is a natural trade-off between accuracy and privacy. The new ability to quantify absolute privacy in addition to accuracy enables the tailoring of the accuracy-privacy ratio to the application at hand. To do this, we consider the randomized parameter *Z*, which defines the degree of randomized strengthening in Genomator’s constraints, see Method Section 2.2. We show how increasing the randomization affects accuracy and privacy.

To demonstrate the approach, we randomly selected 400 SNPs from 400 distinct samples of the 65K SNP dataset, run Genomator to create a synthetic datum and then use Reverse Genomator to randomly reconstruct 500 distinct plausible input sets (of size 400 samples) that could have been used to create the synthetic data. If an individual is part of all reconstructions then the person is at risk of being ‘privacy exposed’. To evaluate the widest range of *Z* parameter values we set the cluster size *N* = 100, and *L* = 0 to isolate the effect of one randomization factor.

**Figure 7.**
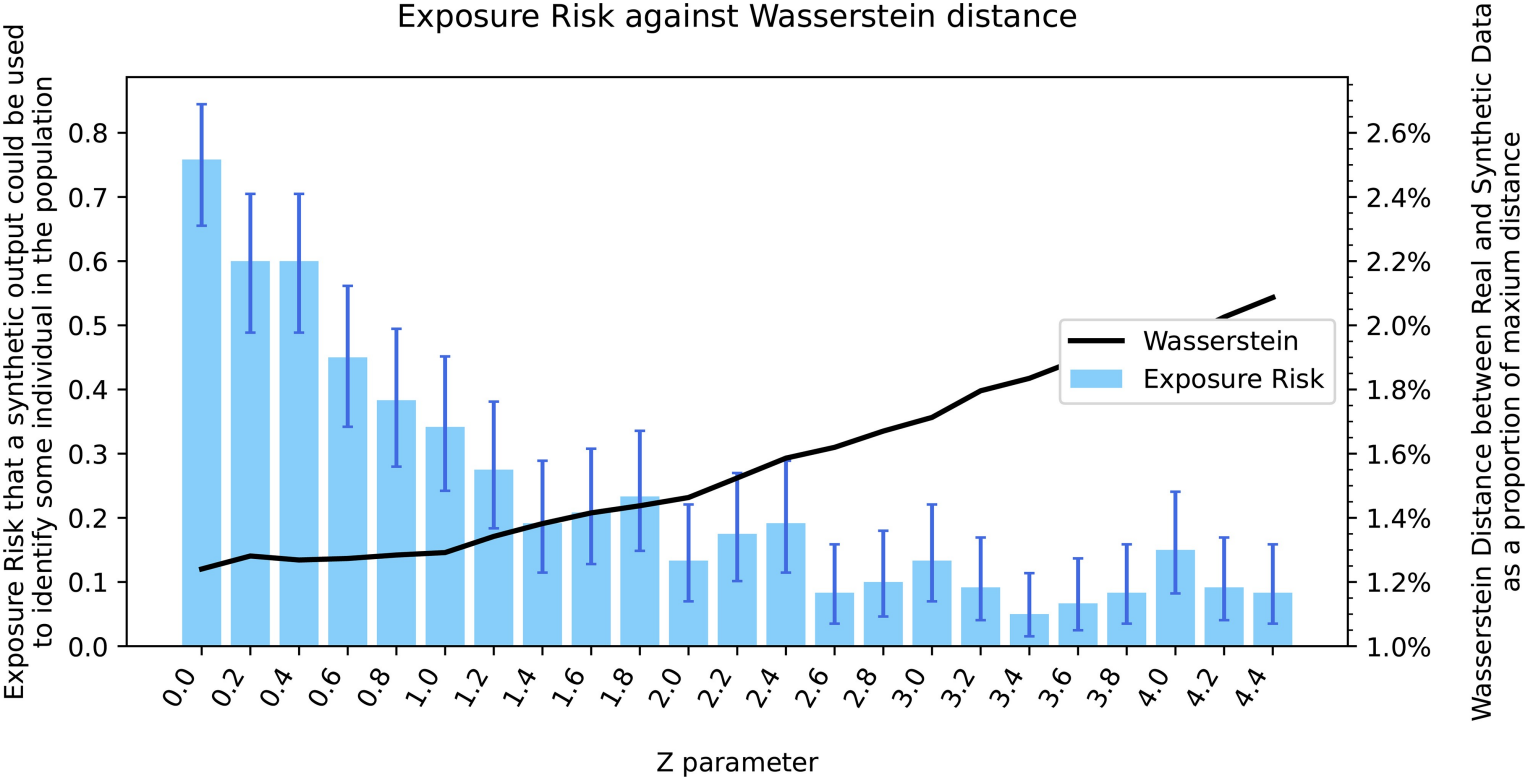
Plot showing the trade-off between privacy and accuracy for Genomator. Shown in blue the experimental likelihood that a synthetic output from Genomator could be used by Reverse Genomator to logically identify any individual in the input dataset (Bernoulli distributed, 90% confidence intervals are shown), across *Z* parameter values for Genomator. Shown in black the Wasserstein distance between synthetic data generated by Genomator for those parameters and the real dataset.

Figure 7 shows that increasing the parameter *Z* lowers the likelihood that any individual is at risk of being privacy exposed, i.e. increases the effective privacy. For example, a y-axis value of 0.1 means that there is a 90% chance that no individual is at risk of being privacy exposed, and thus there is at least a 90% chance that there is no possible deductive reasoning to infer the membership of any individual in the input dataset. We also overlay the sliced-Wasserstein distance between the real and synthetic dataset, visualizing the trade-off between accuracy and privacy. Genomator provides an 8 times improvement in privacy (0.8 to 0.1) when sacrificing 25% accuracy (1.2% versus 1.6%) from *Z* = 0 to *Z* = 2.6.

### 3.5 Utility: Reproduction of well-known pharmacogenetic SNPs in large synthetic datasets

Genomator was used to create a synthetic dataset of 1000 individuals across the 1 313 781 SNPs of the full 3201 samples of chromosomes 10 and 16 from the 1KGP project (Section 2.1). Chromosomes 10 and 16 were chosen as they include three important pharmacogenetic genes and SNPs, namely CYP2C19 (rs4244285 and rs4986893) and VKORC1 (rs9923231). Creating these whole chromosomes was only feasible for Genomator, with WGAN not structured to the dataset, CRBM running out of VRAM due to the large number of samples, and the Markov failing to replicate the subpopulation structure as demonstrated in the PCA results.

Genetic variants in the CYP2C19 gene have been associated with differential metabolism of the antiplatelet drug clopidogrel, where individuals with two loss of function (LOF) alleles have significantly reduced response to the drug (Lee *et al.* 2022). Importantly, the most studied and common LOF CYP2C19 variants, CYP2C19*2 (rs4244285) and CYPC129*3 (rs4986893) have significant varying allele frequencies between ethnicities where both LOF alleles are more frequent in individuals of East Asian compared to those of European and African descents (Fricke-Galindo *et al.* 2016). Similarly, the SNP rs9923231 in the VKORC1 gene is significantly associated with increased warfarin sensitivity and explains the differences of warfarin dosage requirements between ethnicities (Limdi *et al.* 2010).

**Figure 8.**
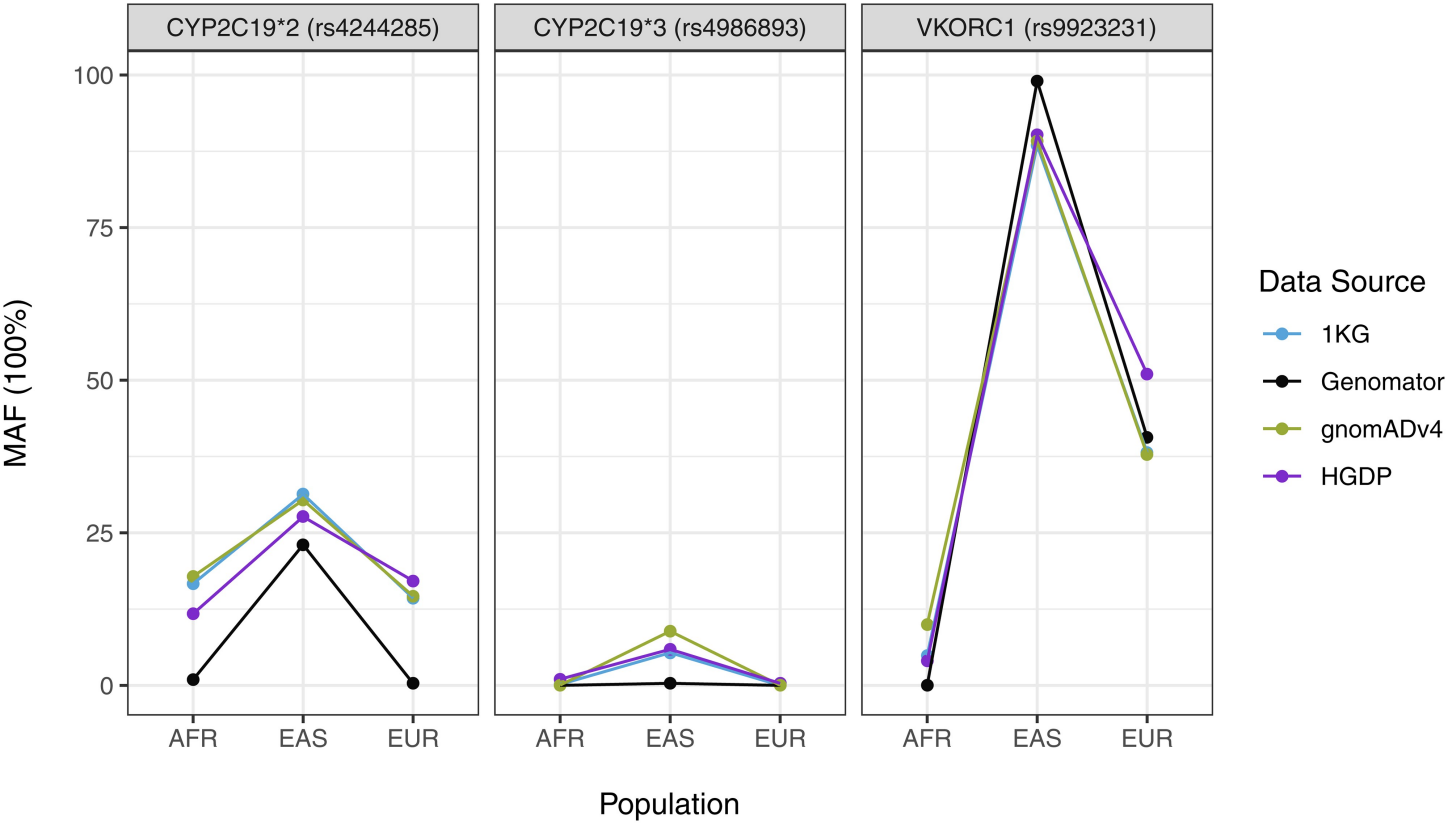
Plot comparing minor allele frequencies of three important pharmacogenetic SNPs (*rs*4244285, *rs*4986893, *rs*9923231) calculated from four data sources: (1) 1000 Genomes Projects (1KG) (*n *= 1668), (2) Genomator generated samples (*n *= 1000), (3) gnomAD v4 (*n *= 682 050), (4) Human Genome Diversity Project (HGDP) (*n *= 526), across three ethnicities [African/African America (AFR), East Asian (EAS), European (EUR)].

Genomator generated samples clustered into four super-population groups based on PCA projection with the 1KGP reference samples: African (AFR), East Asian (EAS), European (EUR), and Others (Fig. 4, available as supplementary data at *Bioinformatics* online, Table 1, available as supplementary data at *Bioinformatics* online). The calculated minor allele frequencies of the three important pharmacogenetic SNPs from Genomator’s generated data were comparable to those of three reference datasets: the 1KGP, gnomAD v4, and the Human Genome Diversity Project (HGDP) across the ethnicities (Fig. 8). Please note that using the default parameters Genomator will not generate SNPs with low MAF (e.g. AFR and EUR samples for rs4986893) as it can compromise the privacy of individuals.

This result shows that Genomator can emulate clinically relevant population-specific allele frequencies at the SNP-level across chromosomes, as well as preserve local genomic structures (LD) and population-specific SNP-frequencies across the whole genome (PCA) as shown in Section 3.1.

## 4 Discussion

While all methods were able to produce genomes that broadly reflect the input data, Genomator effectively captures secondary statistics (e.g. LD) and population structure (e.g. PCA), as it reasons directly over all pairwise associations in its generation process. By contrast, WGAN and CRBM are limited in the amount of information that they can reproduce by the size of their network and hidden layers, and Markov chains are bounded by the chosen haplotype window size in capturing higher-order genome-wide structures.

Genomator can reason over all pairwise associations across the millions of SNPs in the human genome. Unlike other tested methods, where capacity for accuracy degrades as data dimensionality grows, Genomator becomes more constrained and thus accurate as the scale of the input data increases—as revealed by the different slope in runtime results. In this context, Genomator can create whole genomes using a single-thread CPU and minimal RAM, between 3 and 100 times faster at scale.

While all synthetic data can be evaluated for privacy by proxy, to date, only Genomator offers an approach to deduce the absolute privacy of synthetic data directly. This, in turn, allows precision-tuning the accuracy-privacy trade-off to the individual application needs. However, a limitation of Reverse-Genomator is its compute intensity, which limits its application to small segments (∼1000 SNPs) under current computing resources.

Furthermore, while adding privacy bounds can in theory be built into the generation process for GANs (differentially private or DP-GAN) (Xie *et al.* 2018), having an explicit privacy parameter *Z*, directly ensures Genomator does not leak rare SNP pairs and therefore combinations. This parameter is shown to provide a 57%–172% privacy improvement over other methods. Please note that to the best of our knowledge there does not exist a published DP-WGAN or DP-CRBM that can scale to accurately create genomic data. DP could be introduced by adding clipped and bounded noise to the training of WGAN and CRBM. While this may increase privacy, such an approach would have significantly worse accuracy (Tao *et al.* 2021) and runtime than the base-line implementations without the DP-introduced handicap, which were already shown to be outperformed by Genomator (Figs 3 and 4).

We demonstrate the potential in making cohorts accessible to research and the clinic, by creating a representation of a global data resource that contains population-specific information capable of guiding, e.g. pharmacogenetic algorithms for dosage levels of warfarin. It is important to note that ultra-rare variants can have clinically actionable effects and may be important to include in synthetic datasets. However, Genomator attenuates ultra-rare SNPs present in the input dataset, as can be observed when replicating rs4986893 in the AFR and EUR population (Fig. 8). While problematic for replicating ultra-rare variants, this tendency is overall beneficial as it protects against privacy violations for individuals with such private mutations. Section 11, available as supplementary data at *Bioinformatics* online shows that while perfect accuracy can be achieved, it comes at a substantial loss of privacy, i.e. 26% of the synthetic quadruplets can be linked to a single individual, compared to 0.07% for the privacy-preserved setting. While this represents a slight improvement over using the cohort without modification, where 31% of synthetic quadruplets would be linked to a single individual, it disproportionally affects individuals with rare variants or small subpopulations in the dataset. An alternative approach would be to create a privacy-preserved synthetic cohort and randomly spike-in rare SNPs at the expected rate. The sharing of ultra-rare genomic events under privacy considerations hence remains an unsolved issue.

Genomator’s SAT constraints can be extended, such as by adding constraints to avoid leaking disease status, or modified completely to cater to data applications outside the genomic or medical domain. Furthermore, Genomator can benefit from using multiple cores and/or GPU implementation to improve the runtime performance in producing thousands of genomes.

We note that the use of synthetic genomes may be subject to legal and ethical constraints depending on where and how they are used or shared. The requirements and legal responsibilities around synthetic genomes, such as identifying appropriate risk minimization (Rubinstein and Hartzog 2016), and how technology and techniques may interact with applicable regulations and laws (Paltiel *et al.* 2023) may need to be considered.

## 5 Conclusion

In this study, we present Genomator and demonstrate that it is possible to generate synthetic data using a SAT solver to reproduce genomic information. Our technique is accurate, scalable, and computationally efficient as well as configurable to retain the genomic privacy of the individuals in the source dataset. Additionally, we have developed Reverse Genomator to deductively and logically inspect the output from Genomator and calculate the absolute privacy afforded by Genomator. The use of private synthetic data in lieu of real data may allow institutions and biobanks to share genomic information more liberally, advancing health applications and knowledge.

## Supplementary Material

btaf600_Supplementary_Data
