# Supplementary material for: Privacy-hardened and hallucination-resistant synthetic data generation with logic-solvers
Source: Bioinformatics. 2025 Nov 4;41(12):btaf600. doi: 10.1093/bioinformatics/btaf600 (PMC12721864; doi:10.1093/bioinformatics/btaf600)
Supplement: btaf600_Supplementary_Data [file btaf600_supplementary_data.docx]

**Supplemental for: Privacy-hardened and hallucination-resistant synthetic data generation with logic-solvers**

Mark A. Burgess^1^, Brendan Hosking^2^, Roc Reguant^2^, Anubhav Kaphle^3^, Mitchell J. O’Brien^2^, Letitia M.F. Sng^2^, Yatish Jain^2,5^, Denis C. Bauer^4,5,6,7^*

[1] Australian e-Health Research Centre, Commonwealth Scientific and Industrial Research Organisation, Canberra

[2] Australian e-Health Research Centre, Commonwealth Scientific and Industrial Research Organisation, Sydney

[3] Australian e-Health Research Centre, Commonwealth Scientific and Industrial Research Organisation, Melbourne

[4] Australian e-Health Research Centre, Commonwealth Scientific and Industrial Research Organisation, Adelaide

[5] Macquarie University, Applied BioSciences, Faculty of Science and Engineering, Macquarie Park, Australia

[6] University of Sydney, School of Medical Sciences, Department of Biomedical Informatics and Digital Health, Sydney, Australia

[7] The University of Adelaide, Australian Institute for Machine Learning, Adelaide, Australia

## Supplemental Section 1: Description of Genomator Algorithm

Genomator constructs synthetic genome data by formulating a logical problem and solving it using a SAT solver, to make synthetic data that has the properties observed in the real data. Necessarily, the real data (or a subset of it) is considered, and the properties of interest that such real data possess are determined, these properties (or a subset of them) are then transformed into constraints over the process of generating the synthetic data.

Specifically, the properties that Genomator most notably considers are the pairs of genomic variants that do not occur (or only occur below some frequency).

The pairs of genomic variants that exist in the real data are considered, and those pairs of variants that are seen to not exist in the real data are determined. For any pair of variants that do not exist in the real data, a constraint is imposed on the generation of the synthetic data to prevent such a pair from appearing in the synthetic data. Satisfaction of these constraints alone is sufficient to generate realistic synthetic genome data.

This generation is done by constructing a SAT problem and solving it using a SAT solver.

A SAT problem is a class of problem that requires finding the values of a set of variables to satisfy a set of constraints. The Genomator software uses the PySAT library^1^ to interface with a number of available SAT solvers. The background and features of SAT problems and SAT solving approaches are well documented^2^.
In this application of SAT solving, the presence of particular variants are modelled as binary variables, and the constraints that particular pairs of variants cannot co-exist in the synthetic data are modelled as simple Boolean constraints on the values of those binary variables in the SAT problem: of the form “¬x1 OR ¬x2” where x1 and x2 indicate the presence of variants which do not co-occur. The variants that Genomator considers can be configured to be processed in diploid pairs or split into a sequence of haploid singles (the default).

Additional strengthening of the process is facilitated by specifying a ‘privacy’ parameter, a random variable Z. Instead of only considering variant pairs which do not occur in the real data and then adding constraints to prohibit those pairs in the synthetic data, variant pairs which are witnessed to occur in the real data Z times or fewer are considered and seed the generation of constraints prohibiting those pairs in the synthetic data. This additional strengthening directly prohibits rare feature pairs in the real data from occurring in the synthetic data, and has intuitive privacy qualities. We note that Z can be constant or randomised for each pair of variants considered.

Genomator's process of constructing genome data is thus naturally conservative, only producing features and feature pairs that are witnessed in the real data.

The extent to which it is constrained depends on the number of samples (as there are likely fewer pairs of variants not found in a larger dataset of samples) and number of variants (more variants lead to more pairs to consider - and consequently more constraints) in the input dataset, as well as the privacy parameter Z.

To moderate how conservative the resulting synthetic data is, two additional parameters are considered by the algorithm: firstly a random subset of constraints are dropped with probability L, and the size of the number of inputs into the process is specified, N.

The greater the value of Z, the more privacy the process is designed to achieve, but the more conservative and constrained the output and process. Conversely, the greater the value of L and N, the less conservative and constrained the output and process.

The subset of inputs (of size N) is determined by a simple clustering process, creating clusters by nearest Hamming distances.

The process is depicted in Supplementary Figure 1, where a cluster is chosen (of size N), the variant pairs which do not exist in the cluster is determined, a random filtration of probability L is conducted over them, a SAT problem is constructed to generate synthetic data obeying those pairs, and output is determined by a SAT solver. The process is repeated with a random determination of a different cluster to repeatedly generate a range of synthetic data output.

In our experiments described in the paper (except where noted otherwise), we use a cluster size of N=150, set Z to be randomised (Z=0 with probability 2/3, Z=1 with probability 1/3), and set L=0.99. In the results section, we denote ‘Z=α’ to denote Z being a random variable and integer parameter resulting from a rounding down of a uniform random variable in the interval [0,α+1) - noting that by default α =0.5.


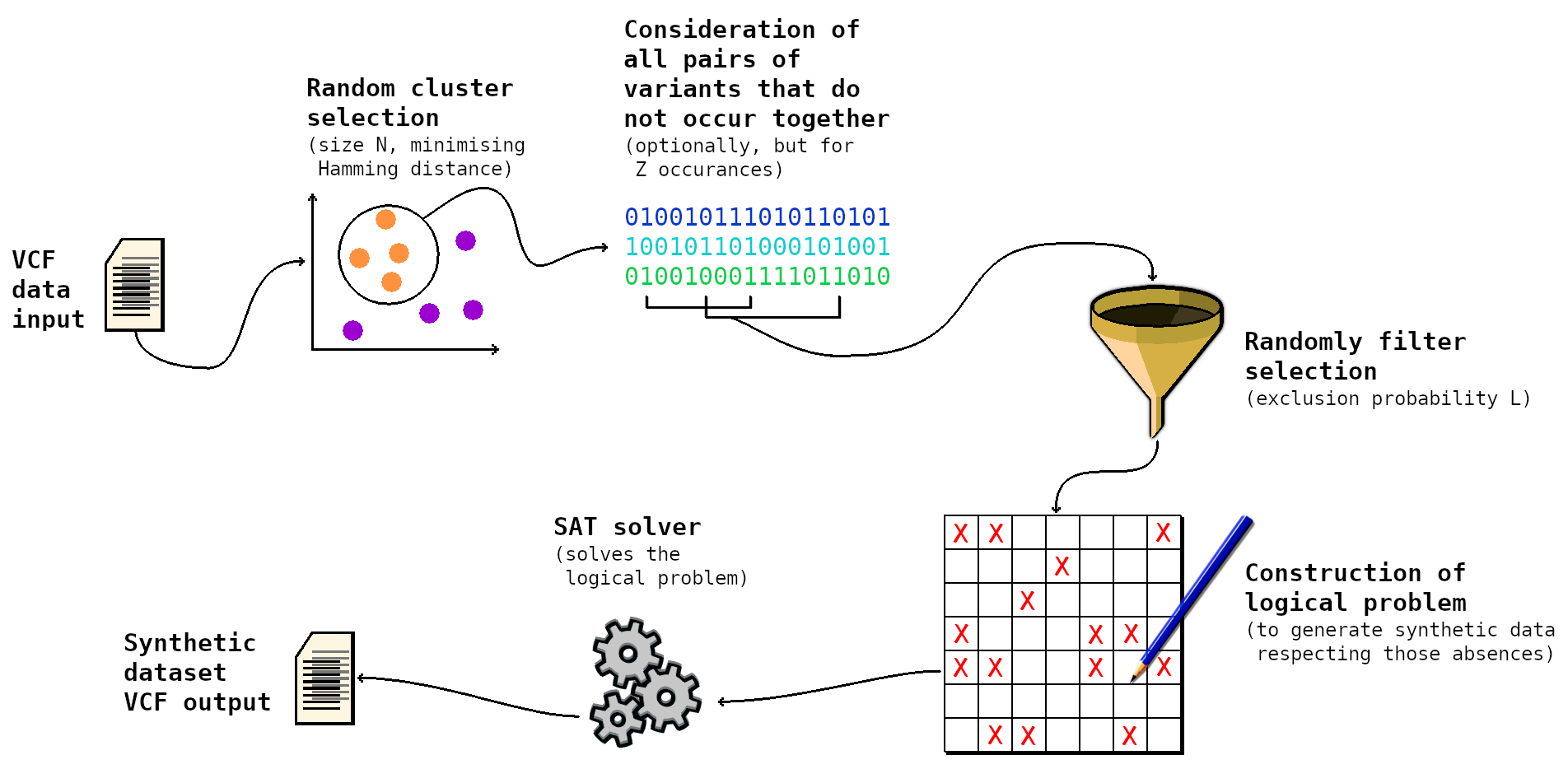
Supplemental Figure 1: Flowchart illustrating the process Genomator uses to construct synthetic genomes, The input data is specified in VCF format, this genomic data is clustered into groups of size N, pairs of variants that do not occur together in the cluster are observed, and a logical problem is generated to construct synthetic data that respects these observations. A SAT solver is used to solve the logical problem and the result - a synthetic genome - is added to a VCF file output; the process is repeated to generate more than one synthetic genome.

## Supplemental Section 2: Description of Reverse Genomator Algorithm

Reverse Genomator conducts Genomator’s logical reasoning in reverse, by constructing a reverse logical problem and solving it using a SAT solver.

As Genomator constructs synthetic genomes by constraints that exclude pairs of variants that do not exist in clusters of the real data, Reverse Genomator reconstructs the plausible clusters of real data such that the pairs of variants that do not exist in these clusters also do not exist in the synthetic data. It is important that the parameters and dataset used by Genomator match those used by Reverse Genomator to give a true indication of the space of logically plausible clusters.
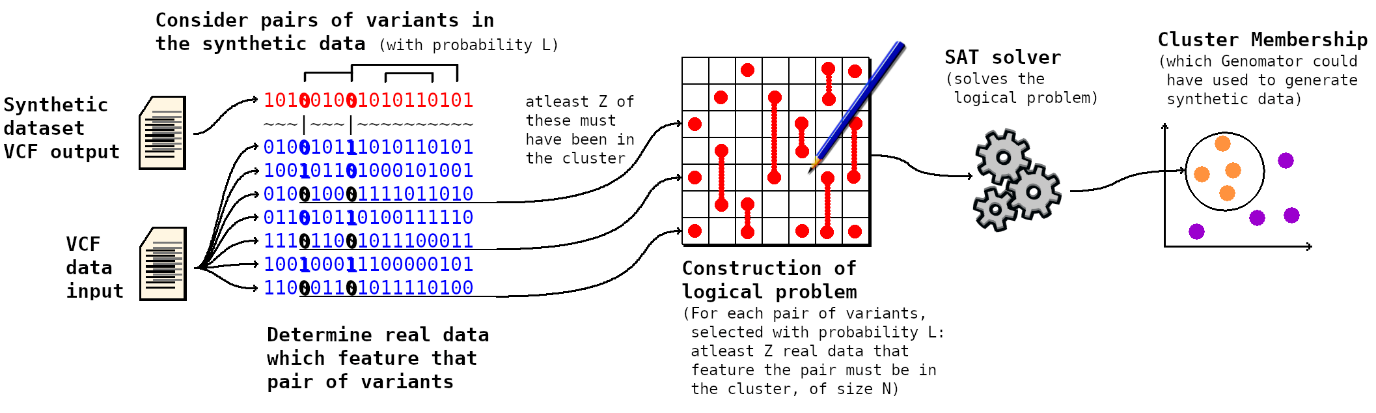
Supplemental Figure 2: Flowchart illustrating the process Reverse Genomator uses to construct plausible input clusters that could have been used by Genomator to generate the synthetic data from the input VCF dataset. For pairs of variants that exist in the synthetic data, selected with probability L, at least a number Z+1 of the real data with that pair of variants must have been in the cluster (which has size N). A SAT solver is used to resolve the plausible clusters.

The membership in these clusters that are resolved by Reverse Genomator then give an indication of the privacy afforded by the synthetic data as, if there is no consensus of membership among the possible clusters, then this gives a demonstration that the synthetic genome could logically have been generated many different ways, with-or-without the presence of any specific genome in the real dataset. Additionally, the preponderance of presence of genomes in these clusters gives an approximation of the *a posteriori* likelihood that those genomes were actually used in the generation of the witnessed output - as demonstrated in Supplemental Theorem 1. And most particularly, if a specific input appears in all the sets resolvable by Reverse Genomator, then it is necessarily in the input cluster actually used.

Privacy considered and measured in this way approximates the Bayesian confidence that an attacker could have about which individuals were used in generating a witnessed output under an extreme scenario where the attacker has access to all the original dataset sequences and only possesses ignorance about which subset was used in the generation of specific synthetic output. In this way Reverse Genomator serves to provide a proxy for the absolute privacy provided by Genomator by deductive reverse inspection of Genomator’s output. We note that this attack model is known as a ‘membership inference attack’ - where an attacker seeks to know if a particular input was part of the input dataset - which contrasts against the ‘attribute inference attack’ as considered in the main paper - where an attacker seeks to infer specific information about a target under the assumption that the individual was part of the input dataset.

**Supplemental Theorem 1:**

Theorem associating the preponderance of counts in Reverse Genomator to the confidence that a hypothetical attacker can have about inputs.


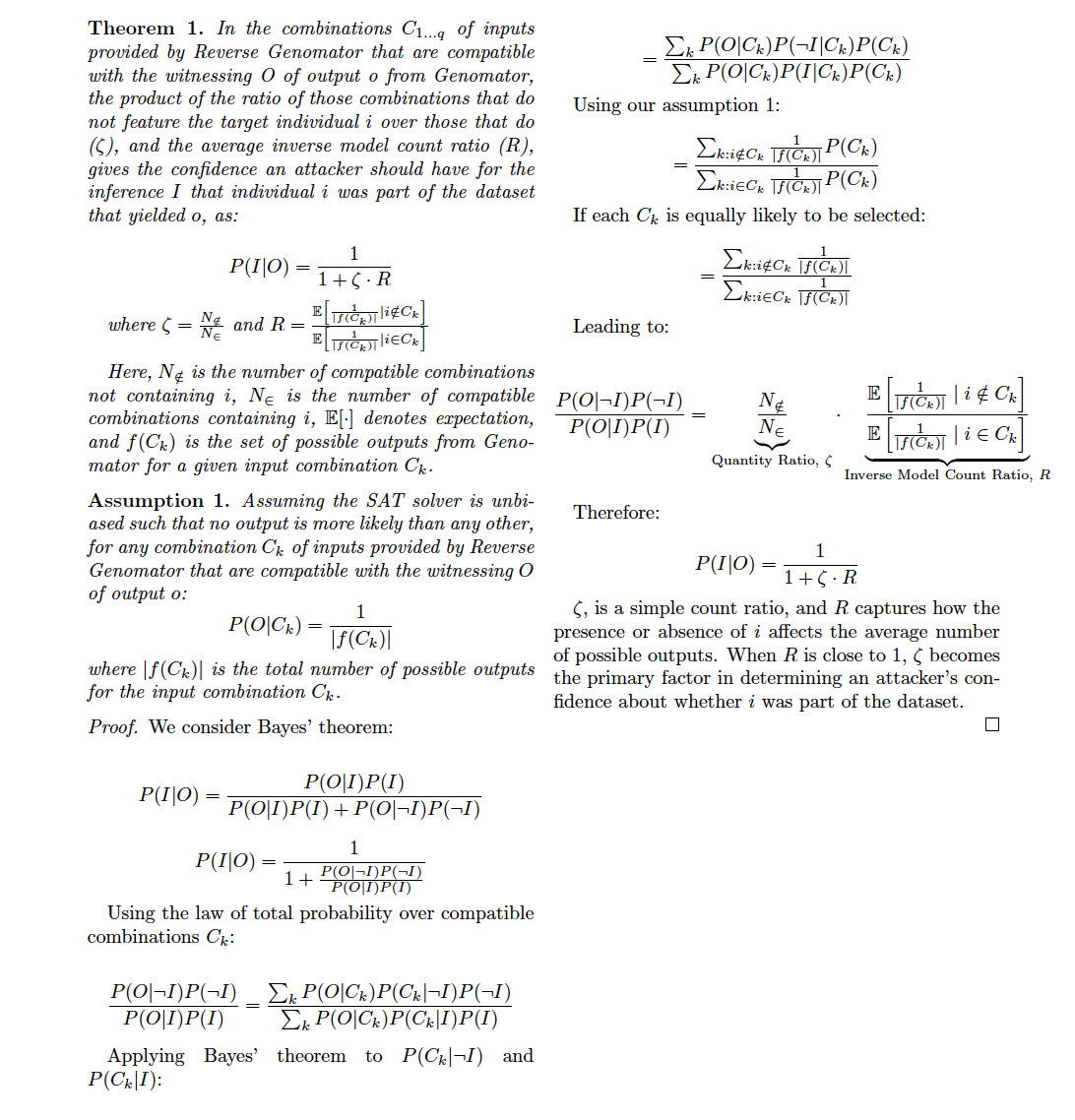


## Supplemental Section 3: Overview of other methods used in the benchmark.

**Supplemental Section 3.1: Markov Chains** are stochastic models of system dynamics where the next state of a system depends on the previous state. In the context of synthetic genome generation, these algorithms stochastically construct the next genome variant based on the previously generated variants in a windowed scan across positions. A primary implementation and analysis of this kind of generation method is given by Samani *et al*^3^. In this context, the next nucleotides are determined by conditional probabilities as measured over the source dataset, and a primary parameter of this method is how many previously generated nucleotides are considered in generating the next, this is called the ‘window size’.

Between different datasets the width of the window is an important parameter to choose. This choice is done to capture the relevant information between important SNPs without making the window so large that tracts of individual specific information get reproduced. Code for this method was adapted from that used in Yelmen *et al.* 2021^4^, with repository <https://gitlab.inria.fr/ml_genetics/public/artificial_genomes>.

**Supplemental Section 3.2: Wasserstein Generative Adversarial Networks** (WGANs) are a bi-modal neural network composed of a generator and a discriminator model. The generator model produces synthetic genomic sequences while the discriminator is trained to evaluate how real the synthetic sequences are. Based on the feedback provided by the discriminator model, the generator model modifies the network’s weights to make the sequences even more indistinguishable, which in-turn then serves to refine the discriminator network.
A Wasserstein GAN (WGAN) is a type of Generative Adversarial Network (GAN) that improves training stability and addresses issues like mode collapse by using the Wasserstein (Earth Mover’s) distance as a loss function instead of the Jensen-Shannon divergence. This is achieved by enforcing a Lipschitz constraint on the discriminator (called the critic) using techniques like weight clipping or gradient penalty. Code for this method was from that used in Yelmen *et al.^5^*, with repository <https://gitlab.inria.fr/ml_genetics/public/artificial_genomes> (retrieved March 2024) Where we note that the models featured layer sizes and connections appropriate for the datasets provided therein, and not compatible with other dataset dimensions.

**Supplemental Section 3.3: Conditional Restricted Boltzmann machine** (CRBMs) are an extension of Restricted Boltzmann machines (RBMs) which are a type of generative neural network model that can learn the underlying probability distributions over a set of input data. They consist of two layers of units: the visible layer which represents the input data and the hidden layer which represents the features/patterns of the input data. The units in each layer are fully connected by weights, which measure how strongly they influence each other. The energy of a joint configuration of visual and hidden units is determined by the sum of activated weights and biases, where the lower the energy the more likely the configuration of the network is. The training process consists of updating these weights via a sampling process with gradient descent (called contrastive divergence), which compares the input data with the generated data and tries to reduce the difference between them. After training, RBMs can be used to generate new data consistent with the learned probability distribution.

Conditional Restricted Boltzmann machines (CRBMs) are an adaptation of RBM architecture considered by Yelmen *et al.* 2023^5^ as a means of more effectively scaling RBM technique to larger genome data. The process consists of training an RBM on an initial section of the training data, and then additional RBMs on consequent sections of data conditional on section of previous data. The CRBM model and code for this method was that used in their paper as found in <https://gitlab.inria.fr/ml_genetics/public/artificial_genomes> (retrieved March 2024).

## Supplemental Section 4: Attribute Inference Experiment Diagram


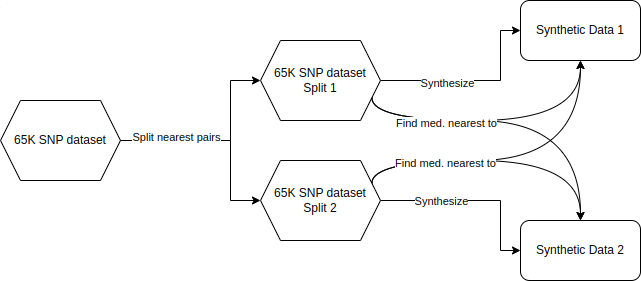


Supplemental Figure 3. The experimental process used in generating a data attribute inference attack scenario. In this context the 65K SNP dataset was split into two equal subsets and synthetic data was generated from each. After this generation the nearest neighbour hamming distance was calculated between each input genome and each of the two synthetic output sets. The median distance from input genomes to the synthetic output set they were used to generate is the ‘in-data’ distance, and the median distance from input genomes to the synthetic output set they were not involved in generating is the ‘out-data’ distance.

## Supplemental Section 5: Reproduction of Well Known Pharmacogenetic SNPs in Large Synthetic Datasets


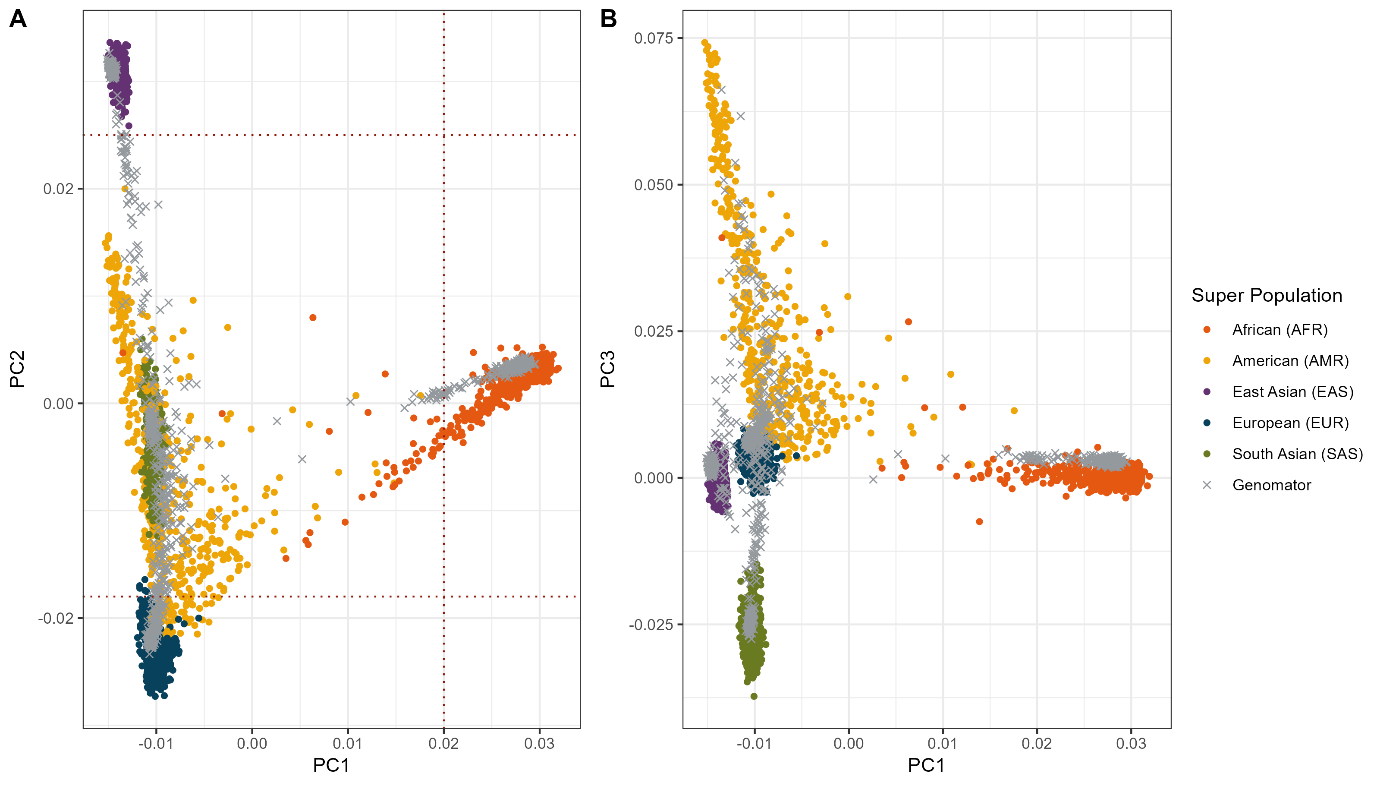


Supplemental Figure 4. Scatterplot of principal components (PC) 1 and 2 (A) and PCs 1 and 3 (B) from the Principal Component Analysis (PCA) of the 1000 Genomes Project (1KG) phase 3 (*n* = 3202), with Genomator generated samples (*n* = 1000) projected onto this space in black crosses. The 1KG samples are annotated according to the corresponding ‘Super Population’ metadata. Red dotted lines denote PC thresholds used to cluster and assign the Genomator generated samples with the 1KG annotated ‘Super Population’ groups.

| Super Population | 1KG (*n*) | 1KG (%) | Genomator (*n*) | Genomator (%) |
| --- | --- | --- | --- | --- |
| African (AFR) | 893 | 27.89 | 267 | 26.7 |
| East Asian (EAS) | 585 | 18.27 | 152 | 15.2 |
| European (EUR) | 633 | 19.77 | 160 | 16 |
| Other (SAS/AMR) | 1091 | 34.07 | 421 | 42.1 |

Supplemental Table 1. Samples from the 1000 Genomes Project (1KG) (*n* = 3202) classified into four ‘Super Populations’ based on their corresponding metadata. Genomator samples (*n* = 1000) were classified into the same four ‘Super Populations’ based on their projection onto principal component analysis space (Supplemental Figure 10).

## Supplemental Section 6: Private vs Fictitious SNP quadruplets

| **METHOD** | **Private** | **- ratio** | **Fictitious** | **- ratio** |
| --- | --- | --- | --- | --- |
| CRBM | 0.507383343177791 | 2.31486 | 0.258010630220197 | 5.35743 |
| Genomator | 0.21918456713415 | 1.0 | 0.0481593707691316 | 1.0 |
| Markov | 0.597673031026253 | 2.72680 | 0.211056137419949 | 4.38245 |
| WGAN | 0.345704630343274 | 1.57723 | 0.15813324169924 | 3.28354 |

Supplemental Table 2: Likelihood of private vs fictitious quadruplets produced for each method, and the ratio of those scores over Genomator’s score.

## Supplemental Section 7: Tabular Results for Attribute Inference Experiment

The Attribute Inference Experiment detailed in the main paper consisted of a range of different methods with different parameters and, for each, the in-data distance and difference between the out-data and in-data distances are reported in the following table. The in-data distance is defined as the median distance between the real data points and the nearest synthetic data point in the set generated from a dataset including them, and the out-data distance is defined as the median distance to the nearest synthetic data point in the set generated from a dataset not including them (see Supplemental Figure 3).

| **METHOD** | **OPTION** | **In-data distance** | **Out-data distance minus in-data distance** |
| --- | --- | --- | --- |
| **CRBM** | **Training Epochs** |  |  |
| CRBM | 2001 | 0.225909819180591 | 6.10E-05 |
| CRBM | 4001 | 0.221408407721065 | 0.000190737773708705 |
| CRBM | 6001 | 0.220073243305104 | 0.000885023270008389 |
| CRBM | 8001 | 0.222087434195468 | 0.00125886930647748 |
| CRBM | 10001 | 0.222529945830472 | 0.00132753490501261 |
| CRBM | 12001 | 0.219195849546044 | 0.000862134737163345 |
| CRBM | 14001 | 0.221927214465553 | 0.00102235446707868 |
| CRBM | 16001 | 0.223529411764706 | 0.00111390859845883 |
| CRBM | 18001 | 0.222362096589609 | 0.00132753490501261 |
| CRBM | 20001 | 0.22410925459678 | 0.00119783321889066 |
| **Genomator** | **Cluster Size, α** |  |  |
| Genomator | 50,0.5 | 0.168215457389181 | 0.00225070572976271 |
| Genomator | 75,0.75 | 0.174631876096742 | 0.00164797436484324 |
| Genomator | 100,1.0 | 0.178873884184024 | 0.00110627908751049 |
| Genomator | 125,1.25 | 0.179049362935836 | 0.000846875715266648 |
| Genomator | 150,1.5 | 0.181322957198444 | 0.000656137941557944 |
| Genomator | 175,1.75 | 0.18226138704509 | 0.000724803540093078 |
| Genomator | 200,2.0 | 0.183535515373465 | 0.000587472343022838 |
| Genomator | 225,2.25 | 0.183199816891737 | 0.000457770656900919 |
| Genomator | 250,2.5 | 0.183016708628977 | 0.000457770656900891 |
| **WGAN** | **Training Epochs** |  |  |
| WGAN | 40 | 0.545830472266728 | -0.000289921416037231 |
| WGAN | 80 | 0.415762569619287 | -4.58E-05 |
| WGAN | 120 | 0.368863965819791 | -0.000282291905088883 |
| WGAN | 160 | 0.305455100328069 | 0.000999465934233612 |
| WGAN | 200 | 0.231082627603571 | 1.53E-05 |
| WGAN | 240 | 0.210421911955444 | 0.000152590218966964 |
| WGAN | 280 | 0.199893186846723 | 0.000381475547417409 |
| WGAN | 320 | 0.20157167925536 | 6.87E-05 |
| WGAN | 360 | 0.205142290379187 | 0.000274662394140535 |
| WGAN | 400 | 0.204150453955901 | 0.000198367284657053 |
| WGAN | 440 | 0.199649042496376 | 0.000274662394140535 |
| WGAN | 480 | 0.20018310826276 | 0.000152590218966964 |
| WGAN | 520 | 0.197268635080491 | 0.000183108262760356 |
| WGAN | 560 | 0.20836194399939 | -3.05E-05 |
| WGAN | 600 | 0.19496452277409 | 9.16E-05 |
| WGAN | 640 | 0.19510185397116 | -1.53E-05 |
| WGAN | 680 | 0.195681696803235 | -4.58E-05 |
| WGAN | 720 | 0.193522545204852 | 6.87E-05 |
| WGAN | 760 | 0.194171053635462 | 0.000244144350347142 |
| WGAN | 800 | 0.193743801022354 | 0.000106813153276875 |
| WGAN | 840 | 0.190768291752499 | 0.000106813153276875 |
| WGAN | 880 | 0.193148699168383 | 0.000198367284657053 |
| WGAN | 920 | 0.191767757686732 | 8.39E-05 |
| WGAN | 960 | 0.196505683985657 | 0.00016784924086366 |
| WGAN | 1000 | 0.190920881971466 | 0.00016784924086366 |
| WGAN | 1040 | 0.194262607766842 | 1.53E-05 |
| WGAN | 1080 | 0.191180285343709 | -6.10E-05 |
| WGAN | 1120 | 0.191393911650263 | 4.58E-05 |
| WGAN | 1160 | 0.194460975051499 | 0.00016784924086366 |
| WGAN | 1200 | 0.193575951781491 | 7.63E-05 |
| WGAN | 1240 | 0.194323643854429 | 0.00016784924086366 |
| WGAN | 1280 | 0.190142671854734 | 0.000259403372243838 |
| WGAN | 1320 | 0.189234760051881 | 0.000267032883192186 |
| WGAN | 1360 | 0.18826581216144 | 9.16E-05 |
| WGAN | 1400 | 0.190295262073701 | 0.000305180437933927 |
| WGAN | 1440 | 0.190814068818189 | 7.63E-05 |
| WGAN | 1480 | 0.194254978255894 | 0.000236514839398794 |
| WGAN | 1520 | 0.193438620584421 | 0.000221255817502097 |
| WGAN | 1560 | 0.192172121766995 | -3.05E-05 |
| WGAN | 1600 | 0.189646753643091 | 0.000137331197070267 |
| WGAN | 1640 | 0.187548638132296 | 0.000183108262760356 |
| WGAN | 1680 | 0.189585717555505 | 0.000289921416037231 |
| **Markov** | **Window Size** |  |  |
| Markov | 10 | 0.199374380102235 | 0.000106813153276875 |
| Markov | 50 | 0.196139467460136 | 0.000137331197070267 |
| Markov | 90 | 0.19464408331426 | 6.10E-05 |
| Markov | 130 | 0.193049515526055 | 0.000267032883192186 |
| Markov | 170 | 0.191042954146639 | 0.000122072175173571 |
| Markov | 210 | 0.189303425650416 | -3.05E-05 |
| Markov | 250 | 0.188197146562905 | 0.000297550926985579 |
| Markov | 290 | 0.187182421606775 | 0.000442511635004195 |
| Markov | 330 | 0.186892500190738 | 0.000656137941557944 |
| Markov | 370 | 0.185641260395209 | 0.000915541313801782 |
| Markov | 410 | 0.18471046005951 | 0.00110627908751049 |
| Markov | 450 | 0.184206912336919 | 0.00144960708018616 |
| Markov | 490 | 0.183779659723812 | 0.00172426947432669 |
| Markov | 530 | 0.183428702220188 | 0.00175478751812011 |
| Markov | 570 | 0.18286411841001 | 0.00205996795605404 |
| Markov | 610 | 0.182574196993973 | 0.00182345311665524 |
| Markov | 650 | 0.182001983672847 | 0.00240329594872971 |
| Markov | 690 | 0.181696803234913 | 0.00251010910200658 |

Supplemental Table 3: The performance of each of the methods and their parameters on the Attribute Inference Experiment

## Supplemental Section 8: Genomator Runtime scaling experiments

To explore and demonstrate the runtime characteristics of Genomator, we consider the time it takes to generate increasing multiples of genomes from the 25,600 SNP segment considered in our main paper’s runtime experiment, section 3.2. We time how long it takes Genomator to generate 1,10,100,1000 and 10000 genomes. The result is plotted in the following Supplemental Figure 5, which shows linear trend.


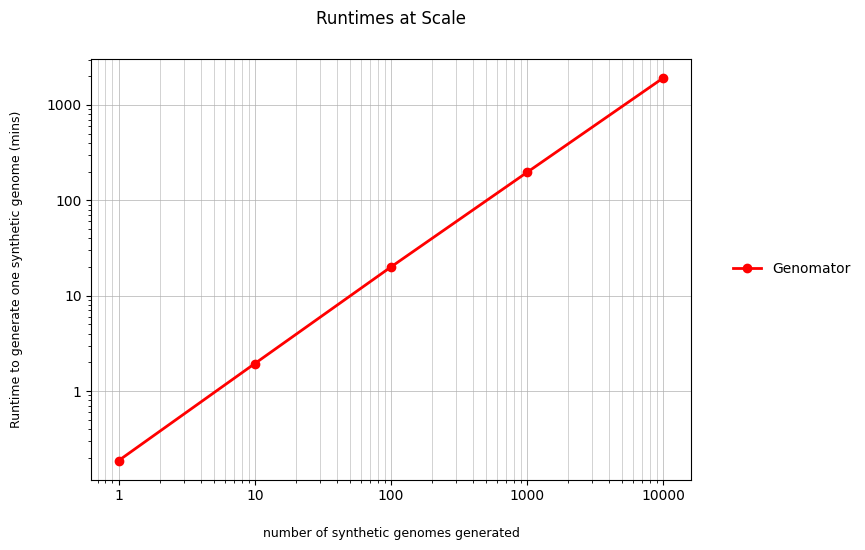


Supplemental Figure 5. Runtime in minutes of Genomator in generating a number of synthetic genomes, of the first 25600 SNP segment of the 1000 genomes project, as considered in main paper section 3.2.

## Supplemental Section 9: Parameter Perturbation Analysis

To consider the effects of varying Genomator’s parameters on accuracy and privacy we ran Genomator with a perturbation of parameters on the 65K SNP dataset of main paper privacy experiment, per section 3.3. The privacy section 3.3 of our main paper shows a Figure 5, which plots a measure of accuracy on X-axis, and a measure of privacy on the Y-axis, tracing out a privacy/accuracy frontier for the methods. We took our parameter point (N=150 Z=1.5 L=0.99) and plotted additional points decreasing and also increasing each of N, Z and L in turn. The resulting figure, showing how changes in these parameters in isolation affect privacy and accuracy is shown as the following Supplemental Figure 6.


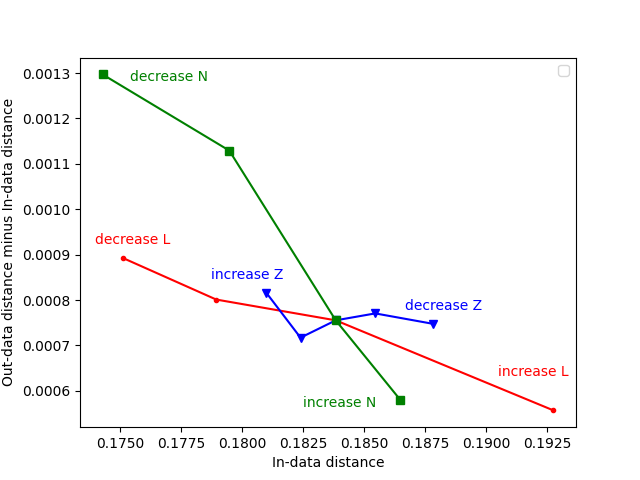

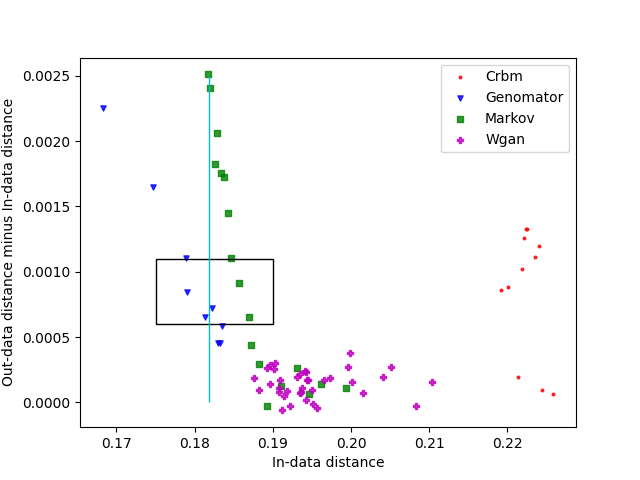


Supplemental Figure 6. The effect of varying Genomator’s parameters N, Z, L on accuracy and privacy as considered in experiment from manuscript section 3.3, together with miniaturised copy of Figure 5 with a rectangle showing the region of interest depicted – for scale.

From this figure we can see that changes in these parameters have a complicated relationship, where an increase or decrease in any one parameter has the effect of increasing accuracy at the cost of privacy, or reversely, increasing privacy at the cost of accuracy. For instance, Increasing L adds more noise to the process, and naturally provides privacy at expense of accuracy. Increasing N makes Genomator consider more genomes at-once in the generation of synthetic data, making the generation process less constrained and more noisy. Increasing Z does add more privacy but not by adding noise, but too high Z can create logical infeasibility. In this context, a judicious choice of the three parameters N,Z,L may be desired to meet user goals (accuracy/privacy) and may be different depending on the structure of the underlying dataset.

## Supplemental Section 10: Parameter Calibration In our main paper we selected parameters N=150,Z=0.5,L=0.99 by a process on the privacy experiment detailed in section 3.3, where we plotted out the privacy/accuracy frontier by a parameter sweep of N and Z, keeping L=0.99. From this parameter sweep we chose the closest point where the median nearest neighbour distance between the synthetic data and the real data was approximately the same median distance from the real data to the real data. To assist others in calibrating these parameters on their own dataset with Genomator, we also added a calibration tool to conduct a similar parameter sweep to identify such a point.

## This tool “experiment_calibrator.py” is found in our repository and given an input VCF datafile will conduct a similar (also configurable) parameter sweep and report the parameters settings that closest satisfy this comparison of medians. We considered application of this parameter sweep in a 2,500-sample cohort from the UKBiobank consisting of 500 self-identified Indian participants, 500 self-identified Chinese participants, 500 self-identified African participants, and 1000 self-identified White British participants. Common (i.e., MAF < 0.1) independent (R^2^ < 0.3) arrayed SNP genotype with 0-missingness were used (i.e., 1337 SNPs). The calibration tool reported the best parameters: N=50,Z=0.5,L=0.99, and to see the working of Genomator with these parameters, we also plotted the PCA of the synthetic data against the UKBiobank data, as shown in the following Supplementary Figure 7. We note that care must be taken, either selecting Genomator’s parameters and/or the ranges of the parameter sweep to ensure the appropriate privacy & accuracy is selected for the user’s context.


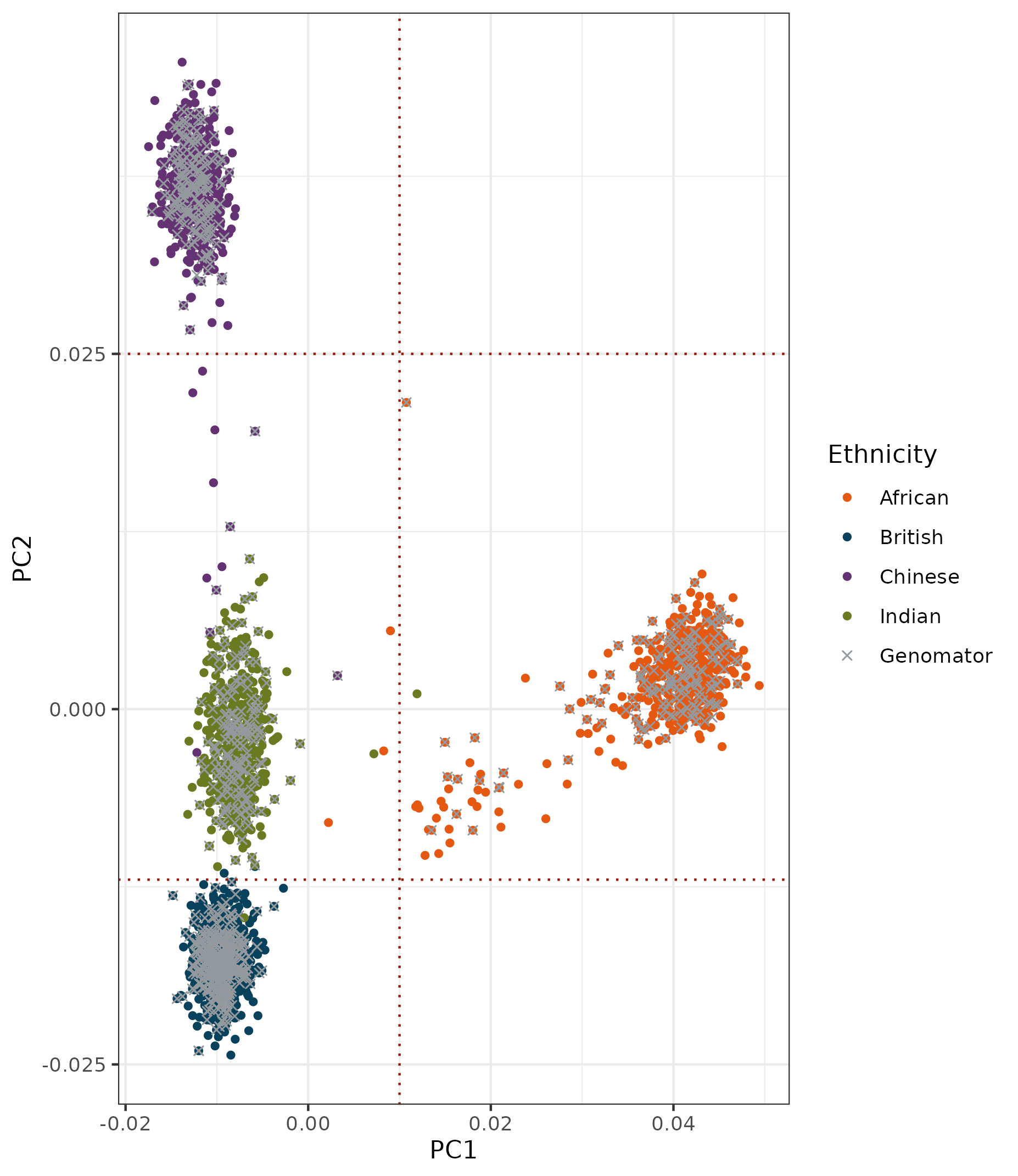


Supplemental Figure 7. PCA of UKbiobank data against synthetic data from Genomator with parameters N=50,Z=0.5,L=0.99.

## Supplemental Section 11: Rare variant analysis with higher accuracy/lower privacy

## To show the effect of decreasing privacy on accuracy of statistics for subpopulations, we considered a configuration of parameters known to give very accurate representation at cost to privacy (N=10,Z=0,L=0). We repeated our experiment per our main paper’s section 3.5, reporting allele frequency of pharmocogenomically relevant genes for subpopulations in generated synthetically generated chromosomes. The results are shown in the following two Supplemental Figures 8 and 9. In this we see that the synthetic data captures the PCA and subpopulation frequency statistics quite well, reproducing some individuals perfectly. This naturally creates accuracy of frequency statistics, and straightforward privacy concerns. To confirm this we ran Quadruplets experiment (as detailed in our paper, section 3.3) on this data between the two Genomator settings (N=150,Z=0.5,L=0.99 and N=10,Z=0,L=0) with results in the following Supplemental Table 4, showing that substantially reduced privacy with the more accurate result – an estimated one quarter of produced synthetic quadruplets being private.


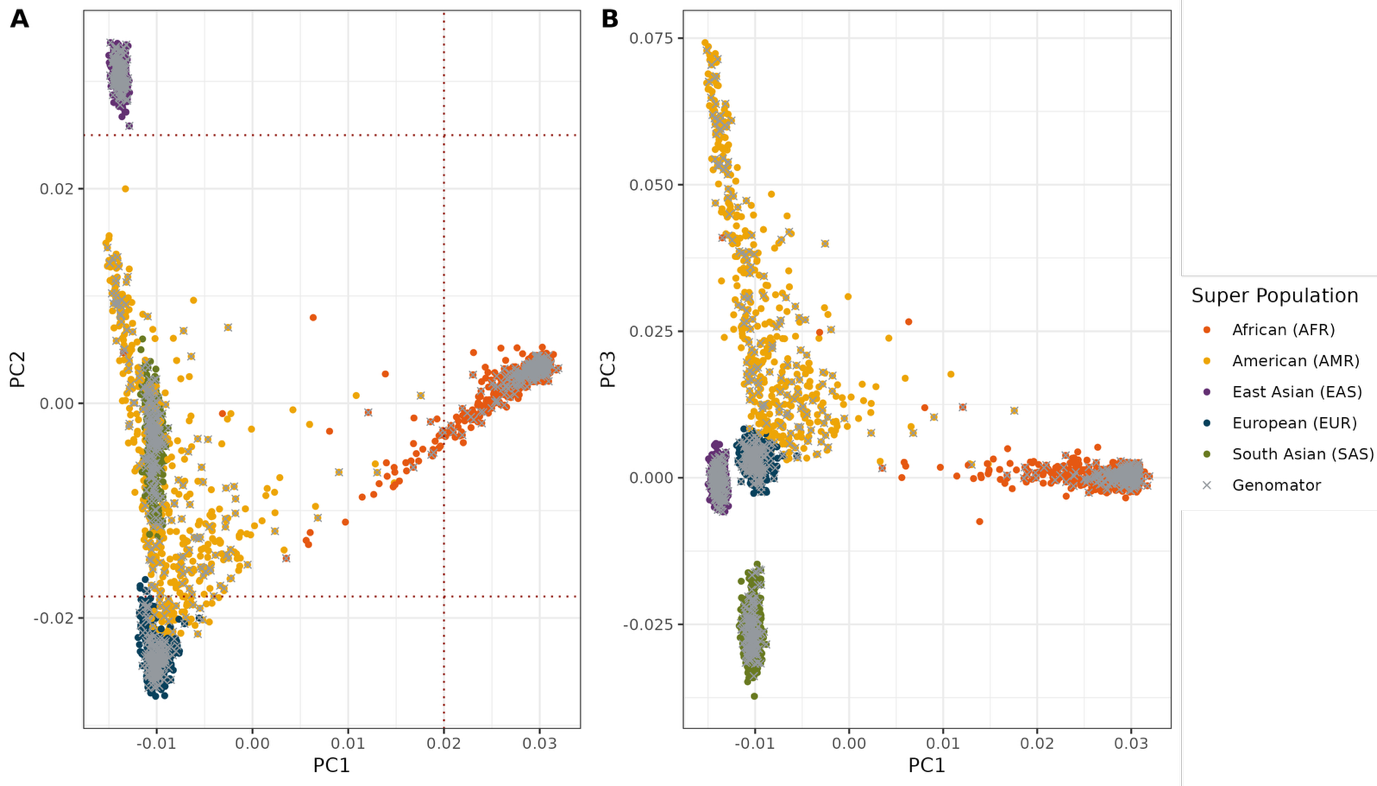


Supplemental Figure 8. Scatterplot of principal components (PC) 1 and 2 (A) and PCs 1 and 3 (B) from the Principal Component Analysis (PCA) of the 1000 Genomes Project (1KG) phase 3 (*n* = 3202), with Genomator generated samples (*n* = 1000, N=10,Z=0,L=0) projected onto this space in black crosses. The 1KG samples are annotated according to the corresponding ‘Super Population’ metadata.


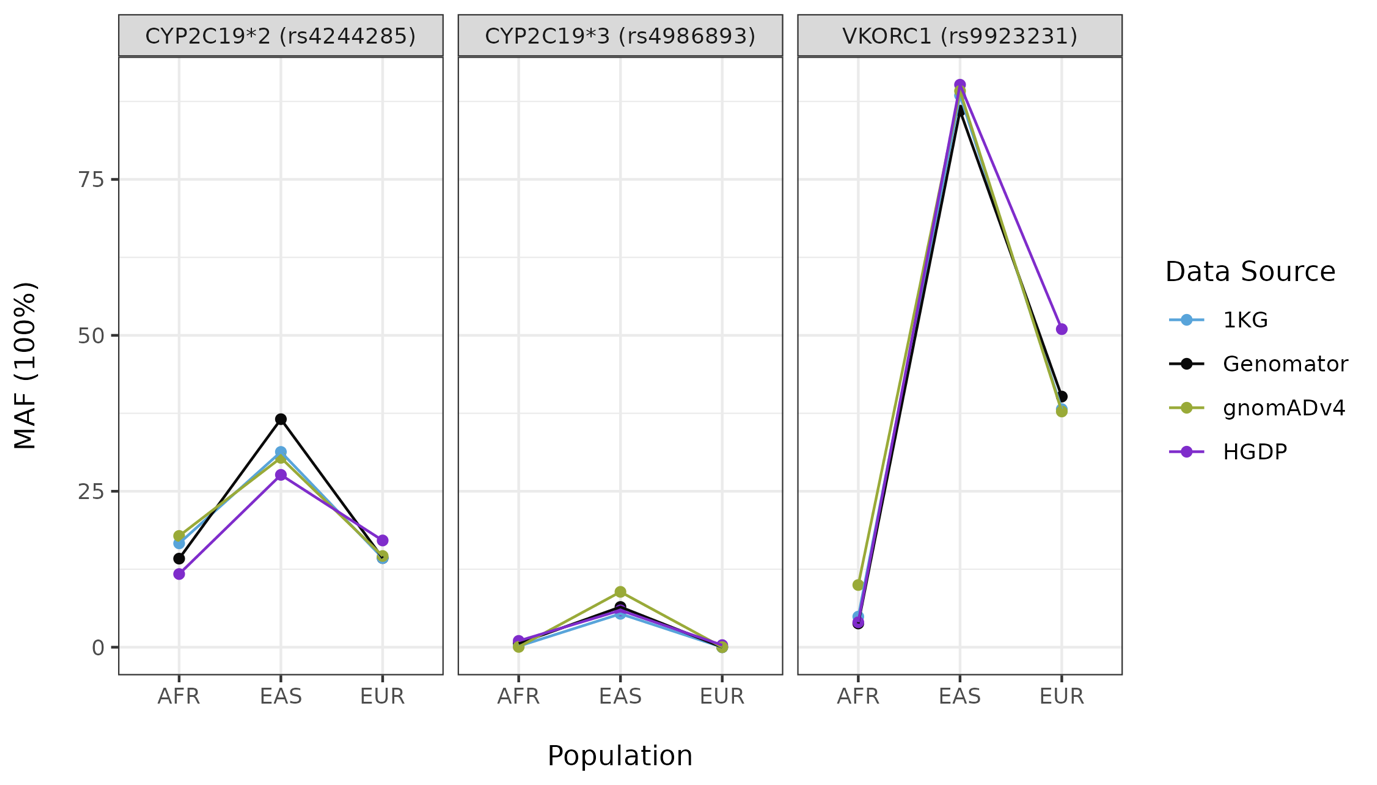
Supplemental Figure 9. Plot comparing minor allele frequencies of three important pharmacogenetic SNPs (rs4244285, rs4986893, rs9923231) calculated from four data sources: (1) 1000 Genomes Projects (1KG) (n = 1,668), (2) Genomator generated samples (n = 1,000, N = 10, Z = 0, L= 0), (3) gnomAD v4 (n = 682,050), (4) Human Genome Diversity Project (HGDP) (n = 526), across three ethnicities (African/African America (AFR), East Asian (EAS), European (EUR)).

| Genomator Parameters | Expectation of a quadruplet appearing in the output being fictitious | Expectation of a quadruplet appearing in the output being private |
| --- | --- | --- |
| N=10, Z=0, L=0 | 0.0 | 0.26401 |
| N=150, Z=0.5, L=0.99 | 0.0001019 | 0.0007297 |
| Random sampling without modification | 0.0 | 0.314171 |

Supplemental Table 4. Results of quadruplets privacy analysis on synthetic chromosomes 10 & 16, showing that more accurate Genomator parameters produces significantly more accurate output, but at cost of privacy.

REFERENCES

1. Ignatiev, A., Morgado, A. & Marques-Silva, J. PySAT: A python toolkit for prototyping with SAT oracles. *Lecture Notes in Computer Science (including subseries Lecture Notes in Artificial Intelligence and Lecture Notes in Bioinformatics)* **10929 LNCS**, 428–437 (2018).

2. Biere, A., Heule, M. & Maaren, H. van. *Handbook of Satisfiability*. (IOS Pres, Washington, DC, 2021).

3. Samani, S. S. *et al.* Quantifying genomic privacy via inference attack with high-order SNV correlations. *Proceedings - 2015 IEEE Security and Privacy Workshops, SPW 2015* 32–40 (2015) doi:10.1109/SPW.2015.21.

4. Yelmen, B. *et al.* Creating artificial human genomes using generative neural networks. *PLoS Genet* **17**, (2021).

5. Yelmen, B. *et al.* Deep convolutional and conditional neural networks for large-scale genomic data generation. *PLoS Comput Biol* **19**, e1011584 (2023).
